# *CYP4B1* polymorphisms and the risk of breast cancer in Chinese women: a case-control study

**DOI:** 10.1186/s12885-023-11477-y

**Published:** 2023-12-01

**Authors:** Yanhai Yin, Liangqian Tong, Zhenling Wan, Yanfang Sui, Fen Li, Qian Huang, Xinhan Zhao

**Affiliations:** 1 Department of Internal Medicine Oncology, The First Affiliated Hospital of Xi’an Jiaotong University School of Medicine, No.76, Yanta West Road, Xi’an City, 710061 Shaanxi Province China; 2 Department of Nuclear Medicine, Hainan General Hospital, Hainan Affiliated Hospital of Hainan Medical University, Haikou, Hainan, 570208 China; 3 Department of Nuclear Medicine, Central South University Xiangya School Affiliated Haikou Hospital, Haikou, Hainan, 570208 China; 4 Department of Pathology, Hainan Women and Children Medical Center, Haikou, Hainan, 570208 China; 5 Department of Rehabilitation Medicine, Central South University Xiangya School Affiliated Haikou Hospital, Haikou, Hainan, 570208 China

**Keywords:** Breast cancer, *CYP4B1*, Single nucleotide polymorphisms, Chinese women

## Abstract

**Background:**

Breast cancer (BC) is one of the malignant diseases threatening the life and health of women worldwide. The *CYP4B1* gene was abnormally expressed in BC and was associated with the prognosis of BC patients. This study aimed to explore the relationship between *CYP4B1* single nucleotide polymorphisms (SNPs) and BC risk in Chinese women.

**Methods:**

A case-control study of 1,143 women (571 patients and 572 healthy individuals) was conducted. Rs2297813 G/T, rs12142787 G/A, and rs3766197 C/T in *CYP4B1* were selected and genotyped by MassARRAY system. The relationships between these SNPs and the risk of BC were assessed by logistic regression analysis. In addition, multi-factor dimensionality reduction (MDR) was used to analyze SNP-SNP interactions.

**Results:**

*CYP4B1* rs2297813 had a risk-increasing effect on BC in women with body mass index (BMI) ≤ 24 kg/m^2^ (OR = 1.72, *p* = 0.026). *CYP4B1* rs12142787 was associated with an increased BC risk in smokers (AA: OR = 1.32, *p* = 0.045). Among non-drinkers, rs2297813 (OR = 1.69, p = 0.009) and rs12142787 (OR = 1.51, p = 0.020) were related to an increased incidence of BC. *CYP4B1* rs3766197 (OR = 1.61*p* = 0.031) was associated with a higher risk of advanced stages (III/IV stage) of BC. Besides, the contributions of *CYP4B1* rs2297813 (OR = 1.55, *p* = 0.021) and rs12142787 (OR = 1.53, *p* = 0.033) to BC risk might be associated with more than one birth in patients with BC. The three-locus model consisting of rs2297813, rs12142787, and rs3766197 was regarded as the best predictive model for BC risk.

**Conclusion:**

*CYP4B1* SNPs were associated with BC risk in Chinese women, especially in patients with BMI ≤ 24 kg/m^2^, smokers, non-drinkers, patients in advanced stages (III/IV stage), and patients who reproduced once. These findings shed light on the relationship between *CYP4B1* SNPs and BC risk in Chinese women.

**Supplementary Information:**

The online version contains supplementary material available at 10.1186/s12885-023-11477-y.

## Background

Breast cancer (BC), one of the most common malignant diseases in women worldwide, can be highly invasive and metastatic [1]. According to Global Cancer Statistics 2020, female breast cancer has surpassed lung cancer as the most commonly diagnosed cancer, with an estimated 2.3 million new cases (11.7%) [2]. Female breast cancer in China accounts for approximately 18% (0.42 million) of the global breast cancer deaths [3]. According to data published in 2022, an estimated 43,250 women died from BC in the United States, in other words, there are nearly 118 BC-related deaths in women per day [4]. And more than 90% of BC-related deaths are related to cancer cell metastasis [5]. Previous studies have shown that breast tumor cells can easily metastasize to some important organs, such as the lung, liver, bone, or brain through blood vessels and lymphatic vessels [1]. BC is an intricate disease characterized by genetic, epigenetic, and phenotypic changes [6]. At present, the carcinogenic mechanism of BC is not fully understood. Some studies have indicated that the occurrence of BC is influenced by genes, lifestyles, and other factors [7–9]. As revealed by investigations, age, family history, obesity, oral contraceptives, status menopausal, smoking, alcohol consumption, lifestyle, and genetics factors are significantly linked to the occurrence and development BC [10], especially genetics factors. Currently, some association studies have identified several susceptible single nucleotide polymorphisms (SNPs) correlated with BC risk [11–13].

Cytochrome P450 (CYP), a superfamily of cysteine heme monooxygenases, is responsible for bio-transforming a large number of endogenous and exogenous substances via oxidative reactions [14]. As one of the CYP isoforms, *CYP4B1*, mainly expressed in lung and bladder tissues can also be detected in BC tissues and cell lines (https://gtexportal.org/home/gene/CYP4B1). Studies have shown that the alteration of *CYP4B1* gene expression may be associated with certain cancers [15], suggesting that *CYP4B1* may work in the development of cancer. The high *CYP4B1* expression increased the chances of bioactivation of carcinogenic aromatic amines, thereby leading to a high risk of cancer growth [15–17]. Gene mutations are associated with protein expression levels, and gene polymorphisms may play a role in disease susceptibility by affecting gene expression and/or enzyme activity [18, 19]. Previous studies have reported that *CYP4B1* polymorphisms are significantly correlated with the risk of various cancers, such as gastric cancer [20], bladder cancer [21], and lung cancer [22]. Iscan et al. have pointed out that *CYP4B1* mRNA is expressed in both breast tumor and tumor-free tissues by reverse transcription-polymerase chain reaction (RT-PCR) [23]. However, to date, little is known about the association of *CYP4B1* SNPs with BC risk.

This case-control study of 1,143 Chinese women was conducted to evaluate the association between *CYP4B1* SNPs and the risk of BC in the Chinese Han population. Additionally, our study revealed the relationship between these SNPs and BC susceptibility by stratified analyses based on multiple factors (demographic and clinicopathological features), which provides a theoretical basis for the prevention and early detection of BC.

## Materials and methods

### Study population and clinical data

In this study, a total of 571 Chinese women diagnosed with BC by clinical examinations and Breast Imaging Reporting and Data System (BI-RADS) were randomly selected as the case group, and 572 healthy Chinese women with no personal or family history of cancer were chosen as the control group. There was no genetic relationship between cases and controls. Patients aged < 20 years or > 70 years with a prior history of cancer, metastatic cancer, radiotherapy, or chemotherapy were excluded. All the healthy volunteers were recruited from the health examination center in the same hospital during the same period as controls. The demographic information (age, weight, height, smoking and drinking habits, reproductive numbers, and menopause) and clinical information (estrogen receptor (ER), progesterone receptor (PR), human epidermal growth factor receptor-2 (HER2), and cell proliferation marker (Ki67), lymph node metastasis (LNM) and staging) about all participates were collected from questionnaires and medical records, respectively. Each participant completed an informed consent form after learning about the purpose of the study. This study was approved by the Ethics Committee of the First Affiliated Hospital of Xi’an Jiaotong University School of Medicine, and all experimental methods were strictly in compliance with the 1964 Helsinki Declaration.

ER, PR, HER2, and cell proliferation marker (Ki67) in patient tissue samples were stained by Immune-histochemical (IHC) assay. According to Breast Cancer, version 3.2018 [24], we diagnosed patients as ER-positive (ER^+^) or ER-negative (ER^−^), PR-positive (PR^+^) or PR-negative (PR^−^), and HER2-positive (HER2^+^) or HER2-negative (HER2^−^). BC cells with more than 25% Ki67 staining were Ki67-positive (Ki67^+^) or otherwise Ki67-negative (Ki67^−^). Tumor staging and lymph node metastasis (LNM) were identified based on the 2010 American Joint Committee on Cancer (AJCC) criteria [25].

### DNA extraction and SNP genotyping

Fasting venous blood samples were collected from all participants in the morning using a vacutainer containing EDTA. Genomic DNA was extracted and purified according to the kit instructions (GoldMag Co., Ltd., Xi’an, China). DNA was then stored in a refrigerator at -80 °C before the experiments. Three SNPs (rs2297813 G/T, rs12142787 G/A, and rs3766197 C/T) of *CYP4B1* were selected based on (1) the variations (113,617) of *CYP4B1* through the e!GRCh37 (http://asia.ensembl.org/Homo_sapiens/Info/Index) database, (2) the variations (923) with global minor allele frequency (MAF) > 0.05, (3) the biallelic variations (402), in which some variations (30) were randomly selected, and (4) combined MassARRAY primer design software, Hardy-Weinberg equilibrium > 0.05, MAF > 0.05 and the call rate > 95% in our study population.,. Furthermore, the MassARRAY system (Agena, San Diego, CA, USA) was applied for genotyping, and all primers were designed by MassARRAY Assay Design software.

### Data analysis

The expression analysis of the *CYP4B1* gene in BC was predicted based on the GEPIA database (http://gepia.cancer-pku.cn/index.html). Kaplan-Meier Plotter database (https://kmplot.com/analysis/) was utilized to assess the correlation between the expression of *CYP4B1* and survival in patients with BC. HaploReg v4.1 (https://pubs.broadinstitute.org/mammals/haploreg/haploreg.php), RegulomeDB (https://regulome.stanford.edu/regulome-search/), and GTEx Portal database (https://gtexportal.org/home/) were applied to predict the potential functions of SNPs.

Data analysis was performed by SPSS software version 21.0 (SPSS, Chicago, IL, USA), and two-sided *p* < 0.05 indicated statistical significance. The differences in demographic characteristics (age, smoking, and drinking) between BC patients and healthy controls were analyzed by *t*-test/*χ*^*2*^-test. HWE was tested by *χ*^*2*^-test to determine whether the subjects in our study reached genetic equilibrium. Logistic regression models adjusted by age, drinking, and smoking were introduced to calculate odds ratios (ORs) and 95% confidence intervals (CIs) with the SNPStats online software (https://www.snpstats.net/start.htm?q=snpstats/start.htm). ORs and CIs represented the effect of *CYP4B1 SNPs* on the risk of BC (OR = 1: no effect; OR < 1: protective factor; OR > 1: risk factor). The association of SNP-SNP interactions with BC risk was evaluated by multi-factor dimensionality reduction (MDR) 3.0.2 software.

## Results

### The characteristics of study participants

The basic characteristics of participants are summarized in Table 1. The average ages of cases and controls were 52.11 ± 10.14 years and 51.88 ± 9.78 years, respectively. And no significant difference in age (*p* = 0.700), smoking (*p* = 0.190), and drinking (*p* = 0.260) between the two groups was detected. Among 571 patients, the majority were menopausal (42.4%), and had more than one reproduction (42.0%). The proportions of early-stage (stage I, II) tumors and late-stage (stage III, IV) tumors were 60.6% and 25.2%, respectively. The results of IHC showed that most cases were diagnosed as ER^+^ (63.4%), PR^+^ (54.8%), and HER2^+^ (22.1%), and had a high level of Ki67 (62.9%). In the BC group, there were 194 patients (34.0%) with LNM and 241 patients (42.2%) without LNM.

**Table 1:** Characteristics of patients with breast cancer and healthy individuals

Characteristics	Group	Case (N = 571)	Control (N = 572)	*p*
Age (years)	Mean ± SD	52.11 ± 10.14	51.88 ± 9.78	0.700^a^
	> 52	270 (47.3%)	271 (47.4%)	
	≤ 52	301 (52.7%)	301 (52.6%)	
Smoking	Yes	292 (51.1%)	270 (47.2%)	0.193^b^
	No	279 (48.9%)	302 (52.8%)	
Drinking	Yes	312 (54.6%)	293 (51.2%)	0.260^b^
	No	259 (45.4%)	279 (48.8%)	
BMI (kg/m^2^)	> 24	95 (16.6%)	139 (24.3%)	
	≤ 24	202 (35.4%)	232 (40.6%)	
	Missing	274 (48.0%)	201 (35.1%)	
Tumor site	Left	210 (36.8%)		
	Right	198 (34.5%)		
Menopause	Yes	242 (42.4%)		
	No	135 (23.6%)		
	Missing	194 (34.0%)		
Reproductive number	= 1	211 (37.0%)		
	> 1	240 (42.0%)		
	Missing	120 (21.0%)		
ER	Positive	362 (63.4%)		
	Negative	161 (28.2%)		
	Missing	48 (8.4%)		
PR	Positive	313 (54.8%)		
	Negative	209 (36.6%)		
	Missing	49 (8.6%)		
Her-2	Positive	126 (22.1%)		
	Negative	194 (34.0%)		
	Missing	251 (44.0%)		
Ki67	> 25%	359 (62.9%)		
	≤ 25%	150 (26.3%)		
	Missing	62 (10.9%)		
LNM	Yes	194 (34.0%)		
	No	241 (42.2%)		
	Missing	136 (23.8%)		
Staging	III, IV	144 (25.2%)		
	I, II	346 (60.6%)		
	Missing	81 (14.2%)		

a: Student’s t test (t test); b: Chi-square test (χ^2^ test)

BMI: Body mass index; ER: Estrogen receptor; PR: Progesterone receptor; Her-2: Human epidermal growth factor receptor 2; LNM: Lymph node metastasis

***CYP4B1*gene expression and its correlation with the prognosis of BC patients and SNP genotype*in silico***.

**Fig. 1:**
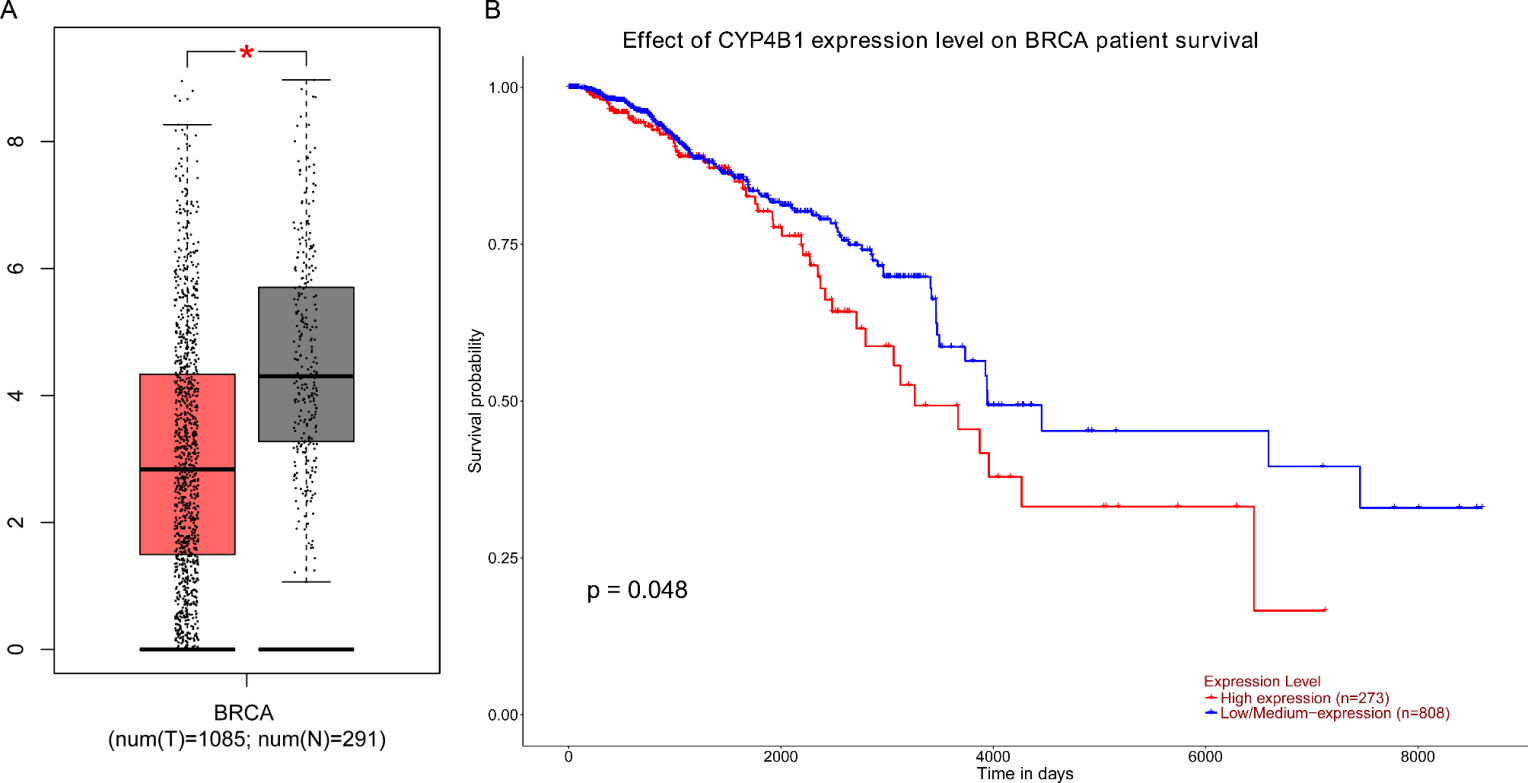
**The expression of*CYP4B1*mRNA in BC tissue (A) and its correlation with prognosis of BC patients (B).** Data from GEPIA database (http://gepia.cancer-pku.cn/index.html) and Kaplan-Meier Plotter database (https://kmplot.com/analysis/). *P < 0.01. Red color means BC tissues and grey color means normal tissues

The expression of the *CYP4B1* gene in normal and BC tissues was predicted by the GEPIA database indicating that the expression level of *CYP4B1* in BC tissues was significantly lower than that in normal tissues (*p* < 0.05, Fig. 1A). Moreover, the results of the Kaplan-Meier Plotter database analysis displayed that the higher *CYP4B1* expression, the worse survival in BC patients (*p* = 0.048, Fig. 1B).

**Fig. 2:**
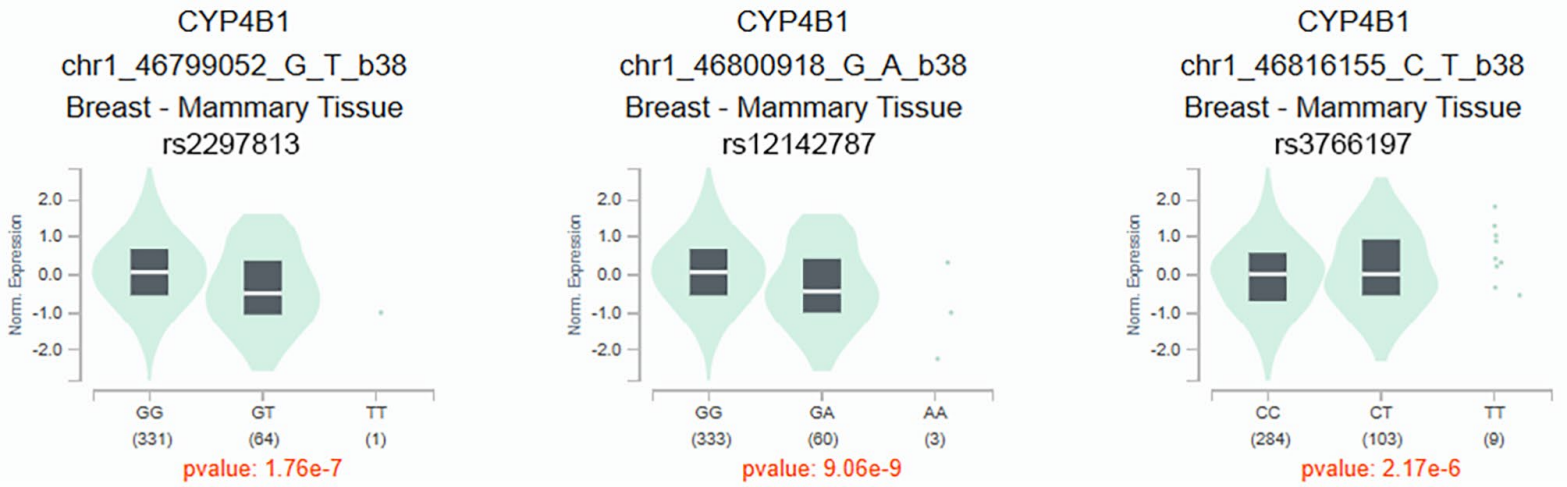
The violin plot for the association between the genotypes of rs2297813, rs12142787, and rs3766197 and the mRNA expression of *CYP4B1* in breast tissues. Data from GTEx Portal database (https://gtexportal.org/home/)

In bioinformatics analysis, these SNPs might be associated with the regulation of promoter/ enhancer histone marks, motif changes, selected eQTL hits, GRASP QTL hits, transcription factor (TF) binding, and chromatin accessibility peak (Table 2). Based on the GTEx Portal database, the genotypes of rs2297813 (*p* = 1.76e-7), rs12142787 (*p* = 9.06e-9), and rs3766197 (*p* = 2.17e-6) were associated with the mRNA expression of *CYP4B1* in breast tissues (Fig. 2). Compared with RS2297813-GG and RS12142787-GG genotype, GT and TT genotypes of rs2297813 and GA and AA genotypes of rs12142787 may be associated with the lower expression of *CYP4B1* mRNA. In addition, the CT and TT genotypes of rs3766197 may have higher mRNA expression of *CYP4B1* than CC genotype.

**Table 2:** The basic information and HWE about the selected SNPs of *CYP4B1*

SNP ID	Chr: position	Alleles	Function	MAF		HWE (*p* value)	Haploreg4.1	RegulomeDB
				Cases	Controls			
rs2297813	1:46799052	G/T	5’-UTR	0.142	0.133	0.469	Promoter histone marks; Enhancer histone marks; DNAse; Motifs changed; Selected eQTL hits	eQTL/caQTL + TF binding + any motif + Footprint + chromatin accessibility peak
rs12142787	1:46800918	G/A	intronic	0.255	0.234	0.295	Promoter histone marks; Enhancer histone marks; Motifs changed; GRASP QTL hits Selected eQTL hits	eQTL/caQTL + TF binding + any motif + Footprint + chromatin accessibility peak
rs3766197	1:46816155	C/T	intronic	0.167	0.166	0.762	Proteins bound; Motifs changed; GRASP QTL hits; Selected eQTL hits	eQTL/caQTL + TF binding / chromatin accessibility peak

*p* < 0.05;

SNP: Single nucleotide polymorphism; Chr: Chromosome; MAF: Minor allele frequency; HWE: Hardy-Weinberg.

HaploReg v4.1 (https://pubs.broadinstitute.org/mammals/haploreg/haploreg.php), RegulomeDB (https://regulome.stanford.edu/regulome-search/)

### Information about candidate SNPs

Basic information about three candidate genetic loci of *CYP4B1* (rs2297813 G/T, rs12142787 G/A, and rs3766197 C/T) is demonstrated in Table 2. The MAFs of each candidate SNP were higher than 0.05 and in line with HWE (*p* > 0.05).

### Association between *CYP4B1* SNPs and BC risk

There was no significant correlation between *CYP4B1* SNPs and BC risk in the overall analysis (Table 3). Further, stratified analysis was conducted to explore the association between CYP4B1 polymorphisms and BC risk in different subgroups based on age, smoking and drinking habits, BMI, and tumor location.

**Table 3:** Analysis of the association between susceptibility of BC and *CYP4B1* SNPs (Overall analysis)

SNP ID	Model	Genotype	Case	Control	Adjusted by age, smoke and drink	
					OR (95% CI)	*p*
rs2297813	Allele	G	980 (85.8%)	992 (86.7%)	1.00	0.530
		T	162 (14.2%)	152 (13.3%)	1.08 (0.85–1.37)	
	Codominant	G/G	418 (73.2%)	432 (75.5%)	1.00	0.430
		G/T	144 (25.2%)	128 (22.4%)	1.17 (0.89–1.54)	
		T/T	9 (1.6%)	12 (2.1%)	0.77 (0.32–1.86)	
	Dominant	G/G	418 (73.2%)	432 (75.5%)	1.00	0.360
		G/T-T/T	153 (26.8%)	140 (24.5%)	1.13 (0.87–1.48)	
	Recessive	G/G-G/T	562 (98.4%)	560 (97.9%)	1.00	0.510
		T/T	9 (1.6%)	12 (2.1%)	0.74 (0.31–1.78)	
	Over-dominant	G/G-T/T	427 (74.8%)	444 (77.6%)	1.00	0.250
		G/T	144 (25.2%)	128 (22.4%)	1.17 (0.89–1.54)	
	Log-additive				1.08 (0.85–1.37)	0.520
rs12142787	Allele	G	846 (74.5%)	874 (76.5%)	1.00	0.250
		A	290 (25.5%)	268 (23.5%)	1.12 (0.92–1.35)	
	Codominant	G/G	316 (55.6%)	339 (59.4%)	1.00	0.410
		A/G	214 (37.7%)	196 (34.3%)	1.18 (0.92–1.51)	
		A/A	38 (6.7%)	36 (6.3%)	1.14 (0.70–1.85)	
	Dominant	G/G	316 (55.6%)	339 (59.4%)	1.00	0.190
		A/G-A/A	252 (44.4%)	232 (40.6%)	1.17 (0.93–1.48)	
	Recessive	G/G-A/G	530 (93.3%)	535 (93.7%)	1.00	0.780
		A/A	38 (6.7%)	36 (6.3%)	1.07 (0.67–1.72)	
	Over-dominant	G/G-A/A	354 (62.3%)	375 (65.7%)	1.00	0.220
		A/G	214 (37.7%)	196 (34.3%)	1.16 (0.91–1.48)	
	Log-additive				1.12 (0.93–1.35)	0.240
rs3766197	Allele	C	951 (83.3%)	951 (83.4%)	1.00	0.930
		T	191 (16.7%)	189 (16.6%)	1.01 (0.81–1.26)	
	Codominant	C/C	399 (69.9%)	395 (69.3%)	1.00	0.580
		T/C	153 (26.8%)	161 (28.2%)	0.94 (0.72–1.22)	
		T/T	19 (3.3%)	14 (2.5%)	1.38 (0.68–2.79)	
	Dominant	C/C	399 (69.9%)	395 (69.3%)	1.00	0.840
		T/C-T/T	172 (30.1%)	175 (30.7%)	0.98 (0.76–1.26)	
	Recessive	C/C-T/C	552 (96.7%)	556 (97.5%)	1.00	0.340
		T/T	19 (3.3%)	14 (2.5%)	1.40 (0.69–2.83)	
	Over-dominant	C/C-T/T	418 (73.2%)	409 (71.8%)	1.00	0.580
		T/C	153 (26.8%)	161 (28.2%)	0.93 (0.72–1.21)	
	Log-additive				1.01 (0.81–1.26)	0.900

95% CI: 95% confidence interval; OR: Odds ratio

*BMI* (Table 4): *CYP4B1-*rs2297813 might be a risk factor for increasing the incidence of BC in participants with BMI ≤ 24 kg/m^2^ under the allele (OR = 1.53, 95% CI 1.01–2.32, *p* = 0.040), dominant (OR = 1.69, 95% CI 1.06–2.69, *p* = 0.027), over-dominant (OR = 1.72, 95% CI 1.07–2.77, *p* = 0.026), and log-additive (OR = 1.56, 95% CI 1.02–2.39, *p* = 0.041) models.

**Table 4:** The SNPs of *CYP4B1* associated with susceptibility of BC in participants (BMI)

SNP ID	Model	Genotype	Case	Control	OR (95% CI)	*p*	Case	Control	OR (95% CI)	*p*
BMI			> 24 kg/m^2^				≤ 24 kg/m^2^			
rs2297813	Allele	G	74 (78.7%)	98 (72.1%)	1.00	0.440	347 (85.9%)	419 (90.3%)	1.00	0.040*
		T	20 (21.3%)	38 (27.9%)	0.73 (0.39–1.37)		57 (14.1%)	45 (9.7%)	1.53 (1.01–2.32)	
	Codominant	G/G	1 (1.1%)	3 (2.2%)	0.37 (0.04–3.90)	0.250	148 (73.3%)	190 (81.9%)	1.00	0.080
		G/T	74 (77.9%)	98 (70.5%)	1.00		51 (25.2%)	39 (16.8%)	1.72 (1.07–2.78)	
		T/T	21 (22.1%)	41 (29.5%)	0.70 (0.38–1.30)		3 (1.5%)	3 (1.3%)	1.24 (0.24–6.38)	
	Dominant	G/G	94 (99.0%)	136 (97.8%)	1.00	0.430	148 (73.3%)	190 (81.9%)	1.00	0.027*
		G/T-T/T	1 (1.1%)	3 (2.2%)	0.41 (0.04–4.23)		54 (26.7%)	42 (18.1%)	1.69 (1.06–2.69)	
	Recessive	G/G-G/T	75 (79.0%)	101 (72.7%)	1.00	0.350	199 (98.5%)	229 (98.7%)	1.00	0.910
		T/T	20 (21.1%)	38 (27.3%)	0.74 (0.40–1.39)		3 (1.5%)	3 (1.3%)	1.10 (0.21–5.66)	
	Over-dominant	G/G-T/T			0.70 (0.40–1.23)	0.210	151 (74.8%)	193 (83.2%)	1.00	0.026*
		G/T	74 (77.9%)	98 (70.5%)	1.00		51 (25.2%)	39 (16.8%)	1.72 (1.07–2.77)	
	Log-additive		20 (21.1%)	38 (27.3%)	0.73 (0.39–1.37)	0.440			1.56 (1.02–2.39)	0.041*
rs12142787	Allele	G	149 (78.4%)	215 (77.3%)	1.00	0.782	301 (74.9%)	365 (79.0%)	1.00	0.150
		A	41 (21.6%)	63 (22.7%)	0.94 (0.60–1.47)		101 (25.1%)	97 (21.0%)	1.26 (0.92–1.74)	
	Codominant	G/G	59 (62.1%)	85 (61.1%)	1.00	0.910	111 (55.2%)	145 (62.8%)	1.00	0.160
		A/G	31 (32.6%)	45 (32.4%)	1.04 (0.59–1.85)		79 (39.3%)	75 (32.5%)	1.49 (0.98–2.24)	
		A/A	5 (5.3%)	9 (6.5%)	0.79 (0.24–2.56)		11 (5.5%)	11 (4.8%)	1.34 (0.55–3.25)	
	Dominant	G/G	59 (62.1%)	85 (61.1%)	1.00	1.000	111 (55.2%)	145 (62.8%)	1.00	0.056
		A/G-A/A	36 (37.9%)	54 (38.9%)	1.00 (0.58–1.72)		90 (44.8%)	86 (37.2%)	1.47 (0.99–2.18)	
	Recessive	G/G-A/G	90 (94.7%)	130 (93.5%)	1.00	0.670	190 (94.5%)	220 (95.2%)	1.00	0.740
		A/A	5 (5.3%)	9 (6.5%)	0.78 (0.25–2.48)		11 (5.5%)	11 (4.8%)	1.16 (0.48–2.77)	
	Over-dominant	G/G-A/A	64 (67.4%)	94 (67.6%)	1.00	0.840	122 (60.7%)	156 (67.5%)	1.00	0.070
		A/G	31 (32.6%)	45 (32.4%)	1.06 (0.60–1.87)		79 (39.3%)	75 (32.5%)	1.45 (0.97–2.18)	
	Log-additive				0.96 (0.62–1.50)	0.870			1.33 (0.96–1.84)	0.089
rs3766197	Allele	C	163 (85.8%)	226 (81.3%)	1.00	0.202	332 (82.2%)	386 (83.2%)	1.00	0.690
		T	27 (14.2%)	52 (18.7%)	0.72 (0.43–1.20)		72 (17.8%)	78 (16.8%)	1.07 (0.75–1.53)	
	Codominant	C/C	68 (71.6%)	91 (65.5%)	1.00	0.150	137 (67.8%)	162 (69.8%)	1.00	0.980
		T/C	27 (28.4%)	44 (31.6%)	0.83 (0.46–1.49)		58 (28.7%)	62 (26.7%)	1.05 (0.68–1.62)	
		T/T	0 (0%)	4 (2.9%)	0.00 (0.00-NA)		7 (3.5%)	8 (3.5%)	0.99 (0.34–2.83)	
	Dominant	C/C	68 (71.6%)	91 (65.5%)	1.00	0.370	137 (67.8%)	162 (69.8%)	1.00	0.850
		T/C-T/T	27 (28.4%)	48 (34.5%)	0.77 (0.43–1.37)		65 (32.2%)	70 (30.2%)	1.04 (0.69–1.58)	
	Recessive	C/C-T/C	95 (100%)	135 (97.1%)	1.00	0.065	195 (96.5%)	224 (96.5%)	1.00	0.960
		T/T	0 (0%)	4 (2.9%)	0.00 (0.00-NA)		7 (3.5%)	8 (3.5%)	0.97 (0.34–2.77)	
	Over-dominant	C/C-T/T	68 (71.6%)	95 (68.3%)	1.00	0.610	144 (71.3%)	170 (73.3%)	1.00	0.830
		T/C	153 (26.8%)	44 (31.6%)	0.93 (0.72–1.21)		58 (28.7%)	62 (26.7%)	1.05 (0.68–1.61)	
	Log-additive				1.01 (0.81–1.26)	0.900			1.03 (0.72–1.46)	0.880

95% CI: 95% confidence interval; OR: Odds ratio

*p* < 0.05: Bold text and ‘*’ represent statistical significance

*Smoking and drinking* (Table 5): When stratified by smoking, rs12142787 was associated with AN increased incidence of BC in smokers under the allele model (OR = 1.32, 95% CI 1.01–1.74, *p* = 0.045). Among non-drinkers rs2297813 (allele: OR = 1.48, 95% CI 1.04–2.10, *p* = 0.029; heterozygote: OR = 1.75, 95% CI 1.16–2.64, *p* = 0.026; dominant: OR = 1.69, 95% CI 1.14–2.52, *p* = 0.009; over-dominant: OR = 1.75, 95% CI 1.16–2.63, *p* = 0.007; log-additive: OR = 1.53, 95% CI 1.06–2.19, *p* = 0.021) and rs12142787 (allele: OR = 1.36, 95% CI 1.03–1.79, *p* = 0.029; dominant: OR = 1.51, 95% CI 1.07–2.13, *p* = 0.020; over-dominant: OR = 1.47, 95% CI 1.03–2.09, *p* = 0.034; log-additive: OR = 1.34, 95% CI 1.02–1.78, *p* = 0.037) were studied to be related to an increased incidence of BC.

**Table 5:** The SNPs of *CYP4B1* associated with susceptibility of breast cancer in participants (Smoking and drinking)

SNP ID	Model	Genotype	Case	Control	OR	*p*	Case	Control	OR	*p*
**Smoking**			**Yes**				**No**			
rs2297813	Allele	G	505 (86.5%)	471 (87.2%)	1.00	0.710	475 (85.1%)	521 (86.3%)	1.00	0.581
		T	79 (13.5%)	69 (12.8%)	1.07 (0.76–1.51)		83 (14.9%)	83 (13.7%)	1.10 (0.79–1.52)	
	Codominant	G/G	217 (74.3%)	207 (76.7%)	1.00	0.540	201 (72%)	225 (74.5%)	1.00	0.780
		G/T	71 (24.3%)	57 (21.1%)	1.19 (0.80–1.77)		73 (26.2%)	71 (23.5%)	1.14 (0.78–1.67)	
		T/T	4 (1.4%)	6 (2.2%)	0.66 (0.18–2.37)		5 (1.8%)	6 (2.0%)	0.93 (0.28–3.13)	
	Dominant	G/G	217 (74.3%)	207 (76.7%)	1.00	0.510	201 (72%)	225 (74.5%)	1.00	0.540
		G/T-T/T	75 (25.7%)	63 (23.3%)	1.14 (0.77–1.68)		78 (28%)	77 (25.5%)	1.12 (0.78–1.63)	
	Recessive	G/G-G/T	288 (98.6%)	264 (97.8%)	1.00	0.480	274 (98.2%)	296 (98%)	1.00	0.870
		T/T	4 (1.4%)	6 (2.2%)	0.63 (0.18–2.27)		5 (1.8%)	6 (2%)	0.90 (0.27–3.02)	
	Over-dominant	G/G-T/T	221 (75.7%)	213 (78.9%)	1.00	0.370	206 (73.8%)	231 (76.5%)	1.00	0.490
		G/T	71 (24.3%)	57 (21.1%)	1.20 (0.81–1.79)		73 (26.2%)	71 (23.5%)	1.14 (0.78–1.67)	
	Log-additive				1.07 (0.76–1.52)	0.690			1.09 (0.78–1.52)	0.610
rs12142787	Allele	G	426 (73.2%)	423 (78.3%)	1.00	0.045*	420 (75.8%)	451 (74.9%)	1.00	0.724
		A	156 (26.8%)	117 (21.7%)	1.32 (1.01–1.74)		134 (24.2%)	151 (25.1%)	0.95 (0.73–1.25)	
	Codominant	G/G	155 (53.3%)	167 (61.9%)	1.00	0.150	161 (58.1%)	172 (57.1%)	1.00	0.890
		A/G	116 (39.9%)	89 (33.0%)	1.38 (0.97–1.96)		98 (35.4%)	107 (35.5%)	1.00 (0.70–1.42)	
		A/A	20 (6.9%)	14 (5.2%)	1.49 (0.73–3.06)		18 (6.5%)	22 (7.3%)	0.85 (0.44–1.65)	
	Dominant	G/G	155 (53.3%)	167 (61.9%)	1.00	0.055	161 (58.1%)	172 (57.1%)	1.00	0.880
		A/G-A/A	136 (46.7%)	103 (38.1%)	1.39 (0.99–1.95)		116 (41.9%)	129 (42.9%)	0.97 (0.70–1.36)	
	Recessive	G/G-A/G	271 (93.1%)	256 (94.8%)	1.00	0.440	259 (93.5%)	279 (92.7%)	1.00	0.620
		A/A	20 (6.9%)	14 (5.2%)	1.32 (0.65–2.67)		18 (6.5%)	22 (7.3%)	0.85 (0.44–1.63)	
	Over-dominant	G/G-A/A	175 (60.1%)	181 (67.0%)	1.00	0.110	179 (64.6%)	194 (64.5%)	1.00	0.920
		A/G	116 (39.9%)	89 (33.0%)	1.33 (0.94–1.88)		98 (35.4%)	107 (35.5%)	1.02 (0.72–1.44)	
	Log-additive				1.30 (0.99–1.71)	0.063			0.96 (0.73–1.25)	0.740
rs3766197	Allele	C	497 (85.1%)	450 (83.6%)	1.00	0.501	454 (81.4%)	501 (83.2%)	1.00	0.407
		T	87 (14.9%)	88 (16.4%)	0.90 (0.65–1.24)		104 (18.6%)	101 (16.8%)	1.14 (0.84–1.54)	
	Codominant	C/C	212 (72.6%)	186 (69.1%)	1.00	0.580	187 (67.0%)	209 (69.4%)	1.00	0.680
		T/C	73 (25.0%)	78 (29.0%)	0.83 (0.57–1.21)		80 (28.7%)	83 (27.6%)	1.07 (0.74–1.54)	
		T/T	7 (2.4%)	5 (1.9%)	1.19 (0.37–3.84)		12 (4.3%)	9 (3.0%)	1.46 (0.60–3.59)	
	Dominant	C/C	212 (72.6%)	186 (69.1%)	1.00	0.390	187 (67.0%)	209 (69.4%)	1.00	0.580
		T/C-T/T	80 (27.4%)	83 (30.9%)	0.85 (0.59–1.23)		92 (33.0%)	92 (30.6%)	1.10 (0.78–1.57)	
	Recessive	C/C-T/C	285 (97.6%)	264 (98.1%)	1.00	0.700	267 (95.7%)	292 (97.0%)	1.00	0.420
		T/T	7 (2.4%)	5 (1.9%)	1.25 (0.39–4.02)		12 (4.3%)	9 (3.0%)	1.44 (0.59–3.50)	
	Over-dominant	C/C-T/T	219 (75.0%)	191 (71.0%)	1.00	0.320	199 (71.3%)	218 (72.4%)	1.00	0.810
		T/C	73 (25.0%)	78 (29.0%)	0.83 (0.57–1.20)		80 (28.7%)	83 (27.6%)	1.05 (0.72–1.51)	
	Log-additive				0.90 (0.65–1.24)	0.510			1.12 (0.83–1.51)	0.460
**Drinking**			**Yes**				**No**			
rs2297813	Allele	G	544 (87.2%)	497 (84.8%)	1.00	0.235	436 (84.2%)	495 (88.7%)	1.00	0.029*
		T	80 (12.8%)	89 (15.2%)	0.82 (0.59–1.14)		82 (15.8%)	63 (11.3%)	1.48 (1.04–2.10)	
	Codominant	G/G	237 (76.0%)	211 (72.0%)	1.00	0.470	181 (69.9%)	221 (79.2%)	1.00	0.026*
		G/T	70 (22.4%)	75 (25.6%)	0.82 (0.56–1.20)		74 (28.6%)	53 (19.0%)	1.75 (1.16–2.64)	
		T/T	5 (1.6%)	7 (2.4%)	0.64 (0.20–2.04)		4 (1.5%)	5 (1.8%)	1.03 (0.27–3.97)	
	Dominant	G/G	237 (76.0%)	211 (72.0%)	1.00	0.240	181 (69.9%)	221 (79.2%)	1.00	0.009*
		G/T-T/T	75 (24.0%)	82 (28.0%)	0.81 (0.56–1.16)		78 (30.1%)	58 (20.8%)	1.69 (1.14–2.52)	
	Recessive	G/G-G/T	307 (98.4%)	286 (97.6%)	1.00	0.490	255 (98.5%)	274 (98.2%)	1.00	0.880
		T/T	5 (1.6%)	7 (2.4%)	0.67 (0.21–2.14)		4 (1.5%)	5 (1.8%)	0.90 (0.24–3.45)	
	Over-dominant	G/G-T/T	242 (77.6%)	218 (74.4%)	1.00	0.330	185 (71.4%)	226 (81.0%)	1.00	0.007*
		G/T	70 (22.4%)	75 (25.6%)	0.83 (0.57–1.21)		74 (28.6%)	53 (19.0%)	1.75 (1.16–2.63)	
	Log-additive				0.81 (0.59–1.13)	0.220			1.53 (1.06–2.19)	0.021*
rs12142787	Allele	G	479 (77.3%)	446 (76.1%)	1.00	0.637	367 (71.1%)	428 (77.0%)	1.00	0.029*
		A	141 (22.7%)	140 (23.9%)	0.94 (0.72–1.23)		149 (28.9%)	128 (23.0%)	1.36 (1.03–1.79)	
	Codominant	G/G	188 (60.6%)	172 (58.7%)	1.00	0.880	128 (49.6%)	167 (60.1%)	1.00	0.064
		A/G	103 (33.2%)	102 (34.8%)	0.93 (0.66–1.31)		111 (43.0%)	94 (33.8%)	1.53 (1.06–2.19)	
		A/A	19 (6.1%)	19 (6.5%)	0.89 (0.46–1.74)		19 (7.4%)	17 (6.1%)	1.42 (0.71–2.87)	
	Dominant	G/G	188 (60.6%)	172 (58.7%)	1.00	0.620	128 (49.6%)	167 (60.1%)	1.00	0.020*
		A/G-A/A	122 (39.4%)	121 (41.3%)	0.92 (0.66–1.27)		130 (50.4%)	111 (39.9%)	1.51 (1.07–2.13)	
	Recessive	G/G-A/G	291 (93.9%)	274 (93.5%)	1.00	0.790	239 (92.6%)	261 (93.9%)	1.00	0.61
		A/A	19 (6.1%)	19 (6.5%)	0.92 (0.47–1.77)		19 (7.4%)	17 (6.1%)	1.20 (0.60–2.37)	
	Over-dominant	G/G-A/A	207 (66.8%)	191 (65.2%)	1.00	0.700	147 (57.0%)	184 (66.2%)	1.00	0.034*
		A/G	103 (33.2%)	102 (34.8%)	0.94 (0.67–1.31)		111 (43.0%)	94 (33.8%)	1.47 (1.03–2.09)	
	Log-additive				0.93 (0.72–1.21)	0.610			1.34 (1.02–1.78)	0.037*
rs3766197	Allele	C	515 (82.5%)	485 (83.3%)	1.00	0.712	436 (84.2%)	466 (83.5%)	1.00	0.770
		T	109 (17.5%)	97 (16.7%)	1.06 (0.78–1.43)		82 (15.8%)	92 (16.5%)	0.95 (0.69–1.32)	
	Codominant	C/C	214 (68.6%)	200 (68.7%)	1.00	0.600	185 (71.4%)	195 (69.9%)	1.00	0.920
		T/C	87 (27.9%)	85 (29.2%)	0.95 (0.67–1.36)		66 (25.5%)	76 (27.2%)	0.93 (0.63–1.38)	
		T/T	11 (3.5%)	6 (2.1%)	1.62 (0.58–4.47)		8 (3.1%)	8 (2.9%)	1.10 (0.40–3.02)	
	Dominant	C/C	214 (68.6%)	200 (68.7%)	1.00	0.980	185 (71.4%)	195 (69.9%)	1.00	0.780
		T/C-T/T	98 (31.4%)	91 (31.3%)	1.00 (0.70–1.41)		74 (28.6%)	84 (30.1%)	0.95 (0.65–1.38)	
	Recessive	C/C-T/C	301 (96.5%)	285 (97.9%)	1.00	0.330	251 (96.9%)	271 (97.1%)	1.00	0.830
		T/T	11 (3.5%)	6 (2.1%)	1.64 (0.60–4.51)		8 (3.1%)	8 (2.9%)	1.12 (0.41–3.07)	
	Over-dominant	C/C-T/T	225 (72.1%)	206 (70.8%)	1.00	0.700	193 (74.5%)	203 (72.8%)	1.00	0.710
		T/C	87 (27.9%)	85 (29.2%)	0.93 (0.65–1.33)		66 (25.5%)	76 (27.2%)	0.93 (0.63–1.37)	
	Log-additive				1.04 (0.77–1.41)	0.780			0.97 (0.70–1.35)	0.870

95% CI: 95% confidence interval; OR: Odds ratio

*p* < 0.05: Bold text and ‘*’ represent statistical significance

*Tumor stage* (Table 6): *CYP4B1* rs3766197 was associated with the higher risk of advanced stages (III/IV stage) of BC under the heterozygote (OR = 1.57, 95% CI 1.04–2.38, *p* = 0.035) and over-dominant (OR = 1.61, 95% CI 1.05–2.46, *p* = 0.031) models.

**Table 6:** The SNPs of *CYP4B1* associated with susceptibility of breast cancer in patients (Tumor staging)

SNP ID	Model	Genotype	Case	Control	OR (95% CI)	*p*
			III, IV	I, II		
rs2297813	Allele	G	238 (82.6%)	600 (86.7%)	1.00	0.099
		T	50 (17.4%)	92 (13.3%)	1.37 (0.94-2.00)	
	Codominant	G/G	108 (75.0%)	250 (72.2%)	1.00	0.130
		G/T	36 (25.0%)	90 (26.0%)	0.92 (0.59–1.45)	
		T/T	0 (0%)	6 (1.7%)		
	Dominant	G/G	108 (75.0%)	250 (72.2%)	1.00	0.520
		G/T-T/T	36 (25.0%)	96 (27.8%)	0.87 (0.55–1.36)	
	Recessive	G/G-G/T	144 (100%)	340 (98.3%)	1.00	0.046
		T/T	0 (0%)	6 (1.7%)	0.00 (0.00-NA)	
	Over-dominant	G/G-T/T	108 (75.0%)	256 (74.0%)	1.00	0.800
		G/T	36 (25.0%)	90 (26.0%)	0.94 (0.60–1.48)	
	Log-additive				0.82 (0.53–1.25)	0.340
rs12142787	Allele	G	210 (73.4%)	514 (74.7%)	1.00	0.676
		A	76 (26.6%)	174 (25.3%)	1.07 (0.78–1.46)	
	Codominant	G/G	85 (59.0%)	185 (53.9%)	1.00	0.340
		A/G	53 (36.8%)	133 (38.8%)	0.87 (0.58–1.33)	
		A/A	6 (4.2%)	25 (7.3%)	0.53 (0.21–1.34)	
	Dominant	G/G	85 (59.0%)	185 (53.9%)	1.00	0.330
		A/G-A/A	59 (41.0%)	158 (46.1%)	0.82 (0.55–1.22)	
	Recessive	G/G-A/G	138 (95.8%)	318 (92.7%)	1.00	0.190
		A/A	6 (4.2%)	25 (7.3%)	0.56 (0.22–1.39)	
	Over-dominant	G/G-A/A	91 (63.2%)	210 (61.2%)	1.00	0.720
		A/G	53 (36.8%)	133 (38.8%)	0.93 (0.62–1.40)	
	Log-additive				0.80 (0.58–1.12)	0.190
rs3766197	Allele	C	238 (82.6%)	576 (83.2%)	1.00	0.820
		T	50 (17.4%	116 (16.8%)	1.04 (0.72–1.50)	
	Codominant	C/C	90 (62.5%)	249 (72.0%)	1.00	0.093
		T/C	49 (34.0%)	85 (24.6%)	1.62 (1.05–2.50)	
		T/T	5 (3.5%)	12 (3.5%)	1.20 (0.41–3.52)	
	Dominant	C/C	90 (62.5%)	249 (72.0%)	1.00	0.035*
		T/C-T/T	54 (37.5%)	97 (28.0%)	1.57 (1.04–2.38)	
	Recessive	C/C-T/C	139 (96.5%)	334 (96.5%)	1.00	0.960
		T/T	5 (3.5%)	12 (3.5%)	1.03 (0.35–2.99)	
	Over-dominant	C/C-T/T	95 (66.0%)	261 (75.4%)	1.00	0.031*
		T/C	49 (34.0%)	85 (24.6%)	1.61 (1.05–2.46)	
	Log-additive				1.39 (0.98–1.97)	0.071

95% CI: 95% confidence interval; OR: Odds ratio

*p* < 0.05: Bold text and ‘*’ represent statistical significance

*Reproductive number* (Table 7): The contribution of *CYP4B1* rs2297813 (allele: OR = 1.55, 95% CI 1.07–2.26, *p* = 0.021; dominant: OR = 1.61, 95% CI 1.05–2.47, *p* = 0.029; over-dominant: OR = 1.55, 95% CI 1.00-2.39, *p* = 0.049; log-additive: OR = 1.58, 95% CI 1.06–2.35, *p* = 0.024) and rs12142787 (allele: OR = 1.38, 95% CI 1.02–1.87, *p* = 0.037; dominant: OR = 1.53, 95% CI 1.03–2.25, *p* = 0.033; log-additive: OR = 1.38, 95% CI 1.01–1.89, *p* = 0.044) to BC risk might be associated with more than one birth in patients with BC.

**Table 7:** The SNPs of *CYP4B1* associated with susceptibility of breast cancer in patients (Reproductive number)

SNP ID	Model	Genotype	Case	Control	OR (95% CI)	*p*
			= 1	> 1		
rs2297813	Allele	G	349 (82.7%)	423 (88.1%)	1.00	0.021*
		T	73 (17.3%)	57 (11.9%)	1.55 (1.07–2.26)	
	Codominant	G/G	142 (67.3%)	185 (77.1%)	1.00	0.080
		G/T	65 (30.8%)	53 (22.1%)	1.57 (1.02–2.43)	
		T/T	4 (1.9%)	2 (0.8%)	2.55 (0.44–14.66)	
	Dominant	G/G	142 (67.3%)	185 (77.1%)	1.00	0.029*
		G/T-T/T	69 (32.7%)	55 (22.9%)	1.61 (1.05–2.47)	
	Recessive	G/G-G/T	207 (98.1%)	238 (99.2%)	1.00	0.340
		T/T	4 (1.9%)	2 (0.8%)	2.28 (0.40-13.03)	
	Over-dominant	G/G-T/T	146 (69.2%)	187 (77.9%)	1.00	0.049*
		G/T	65 (30.8%)	53 (22.1%)	1.55 (1.00-2.39)	
	Log-additive				1.58 (1.06–2.35)	0.024*
rs12142787	Allele	G	300 (71.8%)	372 (77.8%)	1.00	0.037*
		A	118 (28.2%)	106 (22.2%)	1.38 (1.02–1.87)	
	Codominant	G/G	106 (50.7%)	147 (61.5%)	1.00	0.100
		A/G	88 (42.1%)	78 (32.6%)	1.51 (1.01–2.28)	
		A/A	15 (7.2%)	14 (5.9%)	1.59 (0.72–3.53)	
	Dominant	G/G	106 (50.7%)	147 (61.5%)	1.00	0.033*
		A/G-A/A	103 (49.3%)	92 (38.5%)	1.53 (1.03–2.25)	
	Recessive	G/G-A/G	194 (92.8%)	225 (94.1%)	1.00	0.450
		A/A	15 (7.2%)	14 (5.9%)	1.35 (0.62–2.94)	
	Over-dominant	G/G-A/A	121 (57.9%)	161 (67.4%)	1.00	0.071
		A/G	88 (42.1%)	78 (32.6%)	1.44 (0.97–2.15)	
	Log-additive				1.38 (1.01–1.89)	0.044*
rs3766197	Allele	C	356 (84.4%)	394 (82.1%)	1.00	0.362
		T	66 (15.6%)	86 (17.9%)	0.85 (0.60–1.21)	
	Codominant	C/C	150 (71.1%)	165 (68.8%)	1.00	0.220
		T/C	56 (26.5%)	64 (26.7%)	0.90 (0.58–1.38)	
		T/T	5 (2.4%)	11 (4.6%)	0.39 (0.13–1.19)	
	Dominant	C/C	150 (71.1%)	165 (68.8%)	1.00	0.350
		T/C-T/T	61 (28.9%)	75 (31.2%)	0.82 (0.54–1.24)	
	Recessive	C/C-T/C	206 (97.6%)	229 (95.4%)	1.00	0.096
		T/T	5 (2.4%)	11 (4.6%)	0.41 (0.13–1.22)	
	Over-dominant	C/C-T/T	155 (73.5%)	176 (73.3%)	1.00	0.770
		T/C	56 (26.5%)	64 (26.7%)	0.94 (0.61–1.44)	
	Log-additive				0.78 (0.55–1.12)	0.170

95% CI: 95% confidence interval; OR: Odds ratio

*p* < 0.05: Bold text and ‘*’ represent statistical significance

#### Other factors

After analyses stratified based on age and tumor location, no significant association was found (Table S1, *p* > 0.05). Patients were grouped according to menstrual status, tumor biomarkers, and LNM to analyze the relationship between these SNPs and the risk of BC, however, there was no significant correlation between them (Table S2, *p* > 0.05).

### ***CYP4B1*** SNP-SNP interaction in BC risk

**Fig. 3:**
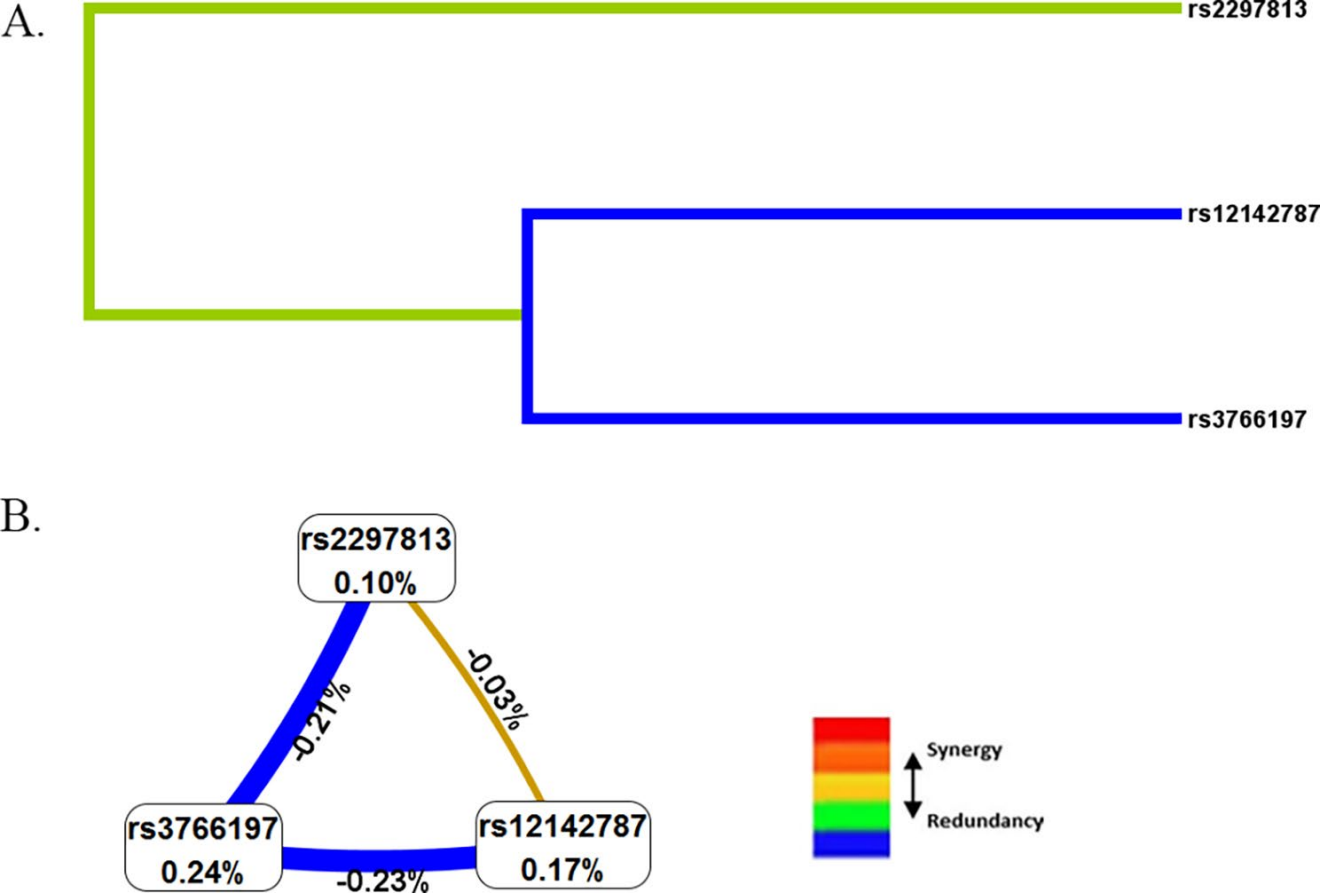
Analysis of SNP-SNP interactions (A. Dendrogram; B. Fruchterman-Reingold. Green and blue represent redundancy or correlation. Values in nodes represent the information gains of individual attribute (main effects). Values between nodes are information gains of each pair of attributes (interaction effects)

MDR was used to analyze and evaluate SNP-SNP interaction (Fig. 3). The blue line indicates that rs12142787 and rs3766197 have a redundant effect on BC risk. As shown in Table 8, the three-locus model (a combination of rs2297813, rs12142787, and rs3766197) had the highest cross-validation consistency (CVC, 10/10) and testing balanced accuracy (Testing Bal. Acc., 0.507), making it the best predictive model for BC risk.

**Table 8:** SNP - SNP interaction models analyzed by the MDR method

Model	Bal.Acc.CV Training	Bal.Acc.CV Testing	OR (95% CI)	*p* value	CV Consistency
rs12142787	0.5201	0.5117	1.18 (0.93–1.49)	0.169	8/10
rs2297813, rs12142787	0.5289	0.5048	1.27 (1.00-1.61)	0.048*	8/10
rs2297813, rs12142787, rs3766197	0.5362	0.5073	1.34 (1.05–1.69)	0.016*	10/10

MDR: Multifactor dimensionality reduction; Bal. Acc.: Balanced accuracy; CVC: Cross-validation consistency; OR: Odds ratio; 95% CI: 95% confidence interval

*p* < 0.05: Bold text and ‘*’ represent statistical significance

## Discussion

BC is a heterogeneous disease, and BC patients with different demographic characteristics and tumor status may exhibit different clinical manifestations [7]. To facilitate diagnosis, prognosis, and treatment, patients are classified according to their clinical data, tumor morphology, genomics, proteomics, and other characteristics [8]. The World Health Organization has identified 21 histological types of BC [26]. Although the mortality of BC shows a trend of decline in recent years, it remains high in all female malignancies [4, 27]. Identifying reliable susceptibility genes associated with BC and investigating the correlation of genetic variants with BC risk is crucial for the effective prevention and treatment of BC [28].

Cytochrome P450 4B1 (CYP4B1) exerts an influence on exogenous biotransformation, and its major extrahepatic expression is associated with some tissue-specific toxicity [16]. CYP4B1 can hydroxylate common endobiotic substrates such as fatty acids, as well as activate xenobiotics such as 4-ipomeanol (4-IPO), perilla ketone (PK) or valproic acid (VPA) [29]. The contribution of *CYP4B1* in cancer is of particular interest as *CYP4B1* gene expression has been found to be altered and presumably associated with certain cancers [30]. In bioinformatics analysis, *CYP4B1* expression in BC tissues was lower than that in normal tissues, and the higher *CYP4B1* expression was correlated with worse survival. Of note, the relationship between *CYP4B1* variants and BC risk has not been reported. Our results displayed that *CYP4B1* rs2297813 was linked to an increased risk of BC in women with BMI ≤ 24 kg/m2, and non-drinkers. And *CYP4B1* rs12142787 had the same effect on BC risk in smokers, and non-drinkers. Moreover, *CYP4B1* rs3766197 was related to the tumor stage, and the association of rs2297813 and rs12142787 with BC risk was related to reproductive number. Bioinformatics analysis showed that these SNPs might be associated with the regulation of promoter/ enhancer histone marks, motif changes, selected eQTL hits, GRASP QTL hits, transcription factor (TF) binding, and chromatin accessibility peak. Based on the GTEx Portal database, the genotypes rs2297813, rs12142787, and rs3766197 were associated with the mRNA expression of *CYP4B1* in breast tissues. These results might suggest that these SNPs may be involved in BC etiology by regulating the expression or function of *CYP4B1*, which provides a theoretical basis for subsequent mechanistic studies.

Because of the clear correlation between increased BMI and increased incidence of BC, obese women have a higher risk of dying from BC than non-obese women [31, 32]. Therefore, the risk of developing BC is strongly affected by BMI. Wang et al. have reported that the SNPs of *NBSI* (rs1805812, rs2735385, and rs6999227), *BRIP1* (rs7220719), and *PTEN* (rs2299941) are associated with BC risk in subjects with BMI < 25 kg/m^2^ [27]. Additionally, *TP53* rs12951053 and *BRIP1* rs16945628 are related to BC risk in people with BMI ≥ 25 kg/m^2^ [27]. Our data revealed the association between *CYP4B1* SNPs and BC risk in patients with BMI ≤ 24 kg/m^2^.

It has been reported that compounds such as polycyclic hydrocarbons contained in tobacco smoke can induce BC [33]. Jones ME et al. have shown that smoking significantly increases the risk of BC in women [34]. In a study by Terry PD et al., the relationship between smoking and BC risk is affected by certain health index of smokers and tumor types, such as the BMI of smokers, and BC patients with ER+/- or PR+/- [33]. Studies over the past few decades have shown positive, negative, or no association between smoking and BC risk [33–35]. Hsieh et al. have reported that rs73229797 can increase the activity of the *CHRNA9* gene promoter and affect the risk of BC in smoking exposure (both smoking and passive smoking) [36]. Furthermore, Naif et al. have also reported that the C and G alleles of *CYP1A* SNPs are associated with the risk of BC in Iraq smokers [37]. Our research is the first to indicate that *CYP4B1* rs12142787 is a risk factor for BC in Chinese female smokers in the allele model. Studies have revealed that smoking status is associated with BC risk [38, 39]. However, the association between rs12142787 and BC risk needs to be further confirmed in subgroup studies based on participants’ smoking status (active or passive).

Reproductive number and tumor stage are closely associated with the progression and metastasis of tumor cells [40, 41]. A previous study has shown that rs2735385 and rs6999227 in *NBS1* are significantly associated with BC in stage III [27]. LincRNA *H19* rs2071095 is associated with BC in stage I [42]. Our results showed that *CYP4B1* rs3766197 was also associated with BC risk according to stratified analysis by tumor stage. *CYP4B1* rs2297813 and rs12142787 were risk factors in the stratified analysis by reproductive number. Yan et al. have reported a remarkable interaction between lncRNA *HOTAIR* and reproductive factors influenced by various factors, such as ER status, menopause, and family history [40, 43]. Our data revealed that *CYP4B1* SNPs were correlated with BC risk after analysis stratified by reproductive numbers.

However, there still are limitations. Firstly, the molecular mechanisms of *CYP4B1* in the occurrence of BC has not been analyzed. In subsequent studies, it is necessary to understand the exact molecular pathway of *CYP4B1* in cells and to investigate the effect of SNPs on *CYP4B1* gene expression. Secondly, the role of these polymorphisms in treatment response and relapse has not been explored, and we will continue to collect samples and refine the experimental design to further explore the role of these polymorphisms in BC treatment response and recurrence. Thirdly, missing data in this study led to errors in some experimental results. Our findings need to be further confirmed in future studies with high-quality and large samples. Nevertheless, to our knowledge, our research remains the first to demonstrate that *CYP4B1* polymorphisms are related to BC risk in different subgroups of Chinese women.

## Conclusion

In summary, *CYP4B1* SNPs could be risk factors for BC in Chinese women, especially in patients with BMI ≤ 24 kg/m^2^, smokers, non-drinkers, patients in advanced stages (III/IV stage), and patients who reproduced once. These findings shed light on the relationship between *CYP4B1* SNPs and BC risk in Chinese women and provide a theoretical and molecular foundation for the prevention of BC in the future.

### Electronic supplementary material

Below is the link to the electronic supplementary material.

Supplementary Material 1

## Data Availability

The datasets used and/or analysed during the current study are available from the corresponding author on reasonable request.

## List of abbreviations

BC: Breast cancer
CYP: Cytochrome P450
RT-PCR: reverse transcription-polymerase chain reaction
SNPs: single nucleotide polymorphisms
BI-RADS: Breast Imaging Reporting and Data System
IHC: Immune-histochemical
ER: estrogen receptor
PR: progesterone receptor
HER2: human epidermal growth factor receptor-2
Ki67: cell proliferation marker
ER^+^: ER-positive
ER^-^: ER-negative
PR^+^: PR-positive
PR^-^: PR-negative
HER2^+^: HER2-positive
HER2^-^: HER2-negative
FISH: fluorescence in situ hybridization
Ki67^+^: Ki67-positive
Ki67^-^: Ki67-negative
LNM: lymph node metastasis
AJCC: American Joint Committee on Cancer
ORs: odds ratios
Cis: confidence intervals
MDR: multi-factor dimensionality reduction
