# Supplementary material for: CYP4B1 polymorphisms and the risk of breast cancer in Chinese women: a case-control study
Source: BMC Cancer. 2023 Dec 1;23:1177. doi: 10.1186/s12885-023-11477-y (PMC10693087; doi:10.1186/s12885-023-11477-y)
Supplement: Supplementary file 1 — Supplementary Material 1 [file 12885_2023_11477_MOESM1_ESM.docx]

Table S1 The SNPs of *CYP4B1* associated with susceptibility of breast cancer in participants (Age and tumor site)

| **SNP ID** | **Model** | **Genotype** | **Case** | **Control** | **OR (95% CI)** | ***p*** | **Case** | **Control** | **OR (95% CI)** | ***p*** |
| --- | --- | --- | --- | --- | --- | --- | --- | --- | --- | --- |
| **Age** |  |  | **> 52 years** | | | | **≤ 52 years** | | | |
| rs2297813 | Allele | G | 468 (86.7%) | 472 (87.1%) | 1.00 | 0.840 | 512 (85.0%) | 520 (86.4%) | 1.00 | 0.510 |
|  |  | T | 72 (13.3%) | 70 (12.9%) | 1.04 (0.73-1.48) |  | 90 (15.0%) | 82 (13.6%) | 1.12 (0.81-1.54) |  |
|  | Codominant | G/G | 201 (74.4%) | 205 (75.7%) | 1.00 | 0.850 | 217 (72.1%) | 227 (75.4%) | 1.00 | 0.470 |
|  |  | G/T | 66 (24.4%) | 62 (22.9%) | 1.09 (0.73-1.63) |  | 78 (25.9%) | 66 (21.9%) | 1.24 (0.85-1.80) |  |
|  |  | T/T | 3 (1.1%) | 4 (1.5%) | 0.76 (0.17-3.46) |  | 6 (2.0%) | 8 (2.7%) | 0.78 (0.26-2.30) |  |
|  | Dominant | G/G | 201 (74.4%) | 205 (75.7%) | 1.00 | 0.720 | 217 (72.1%) | 227 (75.4%) | 1.00 | 0.360 |
|  |  | G/T-T/T | 69 (25.6%) | 66 (24.4%) | 1.07 (0.72-1.59) |  | 84 (27.9%) | 74 (24.6%) | 1.19 (0.82-1.71) |  |
|  | Recessive | G/G-G/T | 267 (98.9%) | 267 (98.5%) | 1.00 | 0.700 | 295 (98.0%) | 293 (97.3%) | 1.00 | 0.580 |
|  |  | T/T | 3 (1.1%) | 4 (1.5%) | 0.75 (0.16-3.38) |  | 6 (2.0%) | 8 (2.7%) | 0.74 (0.25-2.18) |  |
|  | Over-dominant | G/G-T/T | 204 (75.6%) | 209 (77.1%) | 1.00 | 0.650 | 223 (74.1%) | 235 (78.1%) | 1.00 | 0.250 |
|  |  | G/T | 66 (24.4%) | 62 (22.9%) | 1.10 (0.74-1.64) |  | 78 (25.9%) | 66 (21.9%) | 1.25 (0.85-1.81) |  |
|  | Log-additive | --- | --- | --- | 1.04 (0.73-1.50) | 0.820 | --- | --- | 1.11 (0.81-1.53) | 0.520 |
| rs12142787 | Allele | G | 407 (75.7%) | 405 (75%) | 1.00 | 0.800 | 439 (73.4%) | 469 (77.9%) | 1.00 | 0.070 |
|  |  | A | 131 (24.3%) | 135 (25%) | 0.97 (0.73-1.48) |  | 159 (26.6%) | 133 (22.1%) | 1.28 (0.98-1.66) |  |
|  | Codominant | G/G | 157 (58.4%) | 156 (57.8%) | 1.00 | 0.960 | 159 (53.2%) | 183 (60.8%) | 1.00 | 0.170 |
|  |  | A/G | 93 (34.6%) | 93 (34.4%) | 1.00 (0.70-1.44) |  | 121 (40.5%) | 103 (34.2%) | 1.35 (0.96-1.89) |  |
|  |  | A/A | 19 (7.1%) | 21 (7.8%) | 0.91 (0.47-1.76) |  | 19 (6.3%) | 15 (5.0%) | 1.46 (0.72-2.99) |  |
|  | Dominant | G/G | 157 (58.4%) | 156 (57.8%) | 1.00 | 0.940 | 159 (53.2%) | 183 (60.8%) | 1.00 | 0.062 |
|  |  | A/G-A/A | 112 (41.6%) | 114 (42.2%) | 0.99 (0.70-1.39) |  | 140 (46.8%) | 118 (39.2%) | 1.36 (0.98-1.89) |  |
|  | Recessive | G/G-A/G | 250 (92.9%) | 249 (92.2%) | 1.00 | 0.770 | 280 (93.7%) | 286 (95.0%) | 1.00 | 0.460 |
|  |  | A/A | 19 (7.1%) | 21 (7.8%) | 0.91 (0.48-1.73) |  | 19 (6.3%) | 15 (5.0%) | 1.30 (0.65-2.62) |  |
|  | Over-dominant | G/G-A/A | 176 (65.4%) | 177 (65.6%) | 1.00 | 0.940 | 178 (59.5%) | 198 (65.8%) | 1.00 | 0.120 |
|  |  | A/G | 93 (34.6%) | 93 (34.4%) | 1.01 (0.71-1.45) |  | 121 (40.5%) | 103 (34.2%) | 1.30 (0.93-1.82) |  |
|  | Log-additive | --- | --- | --- | 0.98 (0.75-1.28) | 0.850 | --- | --- | 1.28 (0.98-1.68) | 0.069 |
| rs3766197 | Allele | C | 452 (83.7%) | 456 (84.8%) | 1.00 | 0.640 | 499 (82.9%) | 495 (82.2%) | 1.00 | 0.760 |
|  |  | T | 88 (16.3%) | 82 (15.2%) | 1.08 (0.78-1.50) |  | 103 (17.1%) | 107 (17.8%) | 0.95 (0.71-1.29) |  |
|  | Codominant | C/C | 189 (70.0%) | 191 (71.0%) | 1.00 | 0.670 | 210 (69.8%) | 204 (67.8%) | 1.00 | 0.680 |
|  |  | T/C | 74 (27.4%) | 74 (27.5%) | 1.02 (0.70-1.49) |  | 79 (26.2%) | 87 (28.9%) | 0.88 (0.61-1.26) |  |
|  |  | T/T | 7 (2.6%) | 4 (1.5%) | 1.75 (0.50-6.09) |  | 12 (4.0%) | 10 (3.3%) | 1.21 (0.51-2.88) |  |
|  | Dominant | C/C | 189 (70.0%) | 191 (71.0%) | 1.00 | 0.780 | 210 (69.8%) | 204 (67.8%) | 1.00 | 0.600 |
|  |  | T/C-T/T | 81 (30.0%) | 78 (29.0%) | 1.06 (0.73-1.53) |  | 91 (30.2%) | 97 (32.2%) | 0.91 (0.64-1.29) |  |
|  | Recessive | C/C-T/C | 263 (97.4%) | 265 (98.5%) | 1.00 | 0.370 | 289 (96.0%) | 291 (96.7%) | 1.00 | 0.600 |
|  |  | T/T | 7 (2.6%) | 4 (1.5%) | 1.74 (0.50-6.03) |  | 12 (4.0%) | 10 (3.3%) | 1.26 (0.53-2.97) |  |
|  | Over-dominant | C/C-T/T | 196 (72.6%) | 195 (72.5%) | 1.00 | 0.990 | 222 (73.8%) | 214 (71.1%) | 1.00 | 0.440 |
|  |  | T/C | 74 (27.4%) | 74 (27.5%) | 1.00 (0.69-1.46) |  | 79 (26.2%) | 87 (28.9%) | 0.87 (0.61-1.24) |  |
|  | Log-additive | --- | --- | --- | 1.09 (0.78-1.52) | 0.620 | --- | --- | 0.96 (0.72-1.29) | 0.790 |
| **Tumor location** |  |  | **Left** | | | | **Right** | | | |
| rs2297813 | Allele | G | 361 (86.0%) | 992 (86.7%) | 1.00 | 0.696 | 339 (85.6%) | 992 (86.7%) | 1.00 | 0.579 |
|  |  | T | 59 (14.0%) | 152 (13.3%) | 1.07 (0.77-1.48) |  | 57 (14.4%) | 152 (13.3%) | 1.10 (0.79-1.52) |  |
|  | Codominant | G/G | 155 (73.8%) | 432 (75.5%) | 1.00 | 0.840 | 143 (72.2%) | 432 (75.5%) | 1.00 | 0.280 |
|  |  | G/T | 51 (24.3%) | 128 (22.4%) | 1.12 (0.77-1.62) |  | 53 (26.8%) | 128 (22.4%) | 1.25 (0.86-1.82) |  |
|  |  | T/T | 4 (1.9%) | 12 (2.1%) | 0.93 (0.30-2.93) |  | 2 (1.0%) | 12 (2.1%) | 0.49 (0.11-2.21) |  |
|  | Dominant | G/G | 155 (73.8%) | 432 (75.5%) | 1.00 | 0.610 | 143 (72.2%) | 432 (75.5%) | 1.00 | 0.370 |
|  |  | G/T-T/T | 55 (26.2%) | 140 (24.5%) | 1.10 (0.77-1.58) |  | 55 (27.8%) | 140 (24.5%) | 1.18 (0.82-1.71) |  |
|  | Recessive | G/G-G/T | 206 (98.1%) | 560 (97.9%) | 1.00 | 0.860 | 196 (99.0%) | 560 (97.9%) | 1.00 | 0.270 |
|  |  | T/T | 4 (1.9%) | 12 (2.1%) | 0.91 (0.29-2.84) |  | 2 (1.0%) | 12 (2.1%) | 0.46 (0.10-2.08) |  |
|  | Over-dominant | G/G-T/T | 159 (75.7%) | 444 (77.6%) | 1.00 | 0.560 | 145 (73.2%) | 444 (77.6%) | 1.00 | 0.220 |
|  |  | G/T | 51 (24.3%) | 128 (22.4%) | 1.12 (0.77-1.62) |  | 53 (26.8%) | 128 (22.4%) | 1.27 (0.87-1.84) |  |
|  | Log-additive | --- | --- | --- | 1.07 (0.78-1.48) | 0.680 | --- | --- | 1.09 (0.78-1.52) | 0.600 |
| rs12142787 | Allele | G | 314 (74.8%) | 874 (76.5%) | 1.00 | 0.467 | 292 (74.1%) | 874 (76.5%) | 1.00 | 0.333 |
|  |  | A | 106 (25.2%) | 268 (23.5%) | 1.10 (0.85-1.43) |  | 102 (25.9%) | 268 (23.5%) | 1.14 (0.88-1.48) |  |
|  | Codominant | G/G | 119 (56.7%) | 339 (59.4%) | 1.00 | 0.760 | 107 (54.3%) | 339 (59.4%) | 1.00 | 0.370 |
|  |  | A/G | 76 (36.2%) | 196 (34.3%) | 1.11 (0.79-1.55) |  | 78 (39.6%) | 196 (34.3%) | 1.28 (0.91-1.80) |  |
|  |  | A/A | 15 (7.1%) | 36 (6.3%) | 1.21 (0.64-2.29) |  | 12 (6.1%) | 36 (6.3%) | 1.06 (0.53-2.11) |  |
|  | Dominant | G/G | 119 (56.7%) | 339 (59.4%) | 1.00 | 0.480 | 107 (54.3%) | 339 (59.4%) | 1.00 | 0.190 |
|  |  | A/G-A/A | 91 (43.3%) | 232 (40.6%) | 1.12 (0.81-1.54) |  | 90 (45.7%) | 232 (40.6%) | 1.24 (0.90-1.73) |  |
|  | Recessive | G/G-A/G | 195 (92.9%) | 535 (93.7%) | 1.00 | 0.640 | 185 (93.9%) | 535 (93.7%) | 1.00 | 0.910 |
|  |  | A/A | 15 (7.1%) | 36 (6.3%) | 1.16 (0.62-2.17) |  | 12 (6.1%) | 36 (6.3%) | 0.96 (0.49-1.89) |  |
|  | Over-dominant | G/G-A/A | 134 (63.8%) | 375 (65.7%) | 1.00 | 0.630 | 119 (60.4%) | 375 (65.7%) | 1.00 | 0.160 |
|  |  | A/G | 76 (36.2%) | 196 (34.3%) | 1.08 (0.78-1.51) |  | 78 (39.6%) | 196 (34.3%) | 1.27 (0.91-1.78) |  |
|  | Log-additive | --- | --- | --- | 1.10 (0.85-1.42) | 0.460 | --- | --- | 1.14 (0.88-1.49) | 0.320 |
| rs3766197 | Allele | C | 356 (84.8%) | 951 (83.4%) | 1.00 | 0.524 | 324 (81.8%) | 951 (83.4%) | 1.00 | 0.464 |
|  |  | T | 64 (15.2%) | 189 (16.6%) | 0.90 (0.66-1.23) |  | 72 (18.2%) | 189 (16.6%) | 1.12 (0.83-1.51) |  |
|  | Codominant | C/C | 150 (71.4%) | 395 (69.3%) | 1.00 | 0.800 | 135 (68.2%) | 395 (69.3%) | 1.00 | 0.350 |
|  |  | T/C | 56 (26.7%) | 161 (28.2%) | 0.91 (0.64-1.31) |  | 54 (27.3%) | 161 (28.2%) | 0.97 (0.67-1.40) |  |
|  |  | T/T | 4 (1.9%) | 14 (2.5%) | 0.76 (0.25-2.35) |  | 9 (4.5%) | 14 (2.5%) | 1.90 (0.80-4.51) |  |
|  | Dominant | C/C | 150 (71.4%) | 395 (69.3%) | 1.00 | 0.550 | 135 (68.2%) | 395 (69.3%) | 1.00 | 0.810 |
|  |  | T/C-T/T | 60 (28.6%) | 175 (30.7%) | 0.90 (0.64-1.28) |  | 63 (31.8%) | 175 (30.7%) | 1.04 (0.74-1.48) |  |
|  | Recessive | C/C-T/C | 206 (98.1%) | 556 (97.5%) | 1.00 | 0.660 | 189 (95.5%) | 556 (97.5%) | 1.00 | 0.150 |
|  |  | T/T | 4 (1.9%) | 14 (2.5%) | 0.78 (0.25-2.40) |  | 9 (4.5%) | 14 (2.5%) | 1.92 (0.81-4.52) |  |
|  | Over-dominant | C/C-T/T | 154 (73.3%) | 409 (71.8%) | 1.00 | 0.650 | 144 (72.7%) | 409 (71.8%) | 1.00 | 0.750 |
|  |  | T/C | 56 (26.7%) | 161 (28.2%) | 0.92 (0.64-1.31) |  | 54 (27.3%) | 161 (28.2%) | 0.94 (0.65-1.36) |  |
|  | Log-additive | --- | --- | --- | 0.90 (0.66-1.23) | 0.510 | --- | --- | 1.11 (0.82-1.50) | 0.490 |

OR: Odds ratio; 95% CI: 95% confidence interval.

Table S2 The SNPs of *CYP4B1* associated with susceptibility of BC in patients (Menstrual status, Tumor biomarkers, and Lymph node metastasis)

| **SNP ID** | **Model** | **Genotype** | **Case** | **Control** | **OR (95% CI)** | ***p*** |
| --- | --- | --- | --- | --- | --- | --- |
| **Menstrual status** |  |  | **Post-menopausal** | **Pre-menopausal** |  |  |
| rs2297813 | Allele | G | 425 (87.8%) | 224 (83.0%) | 1.00 | 0.065 |
|  |  | T | 59 (12.2%) | 46 (17.0%) | 0.68 (0.45-1.03) |  |
|  | Codominant | G/G | 185 (76.5%) | 90 (66.7%) | 1.00 | 0.260 |
|  |  | G/T | 55 (22.7%) | 44 (32.6%) | 0.58 (0.29-1.14) |  |
|  |  | T/T | 2 (0.8%) | 1 (0.7%) | 1.76 (0.04-79.84) |  |
|  | Dominant | G/G | 185 (76.5%) | 90 (66.7%) | 1.00 | 0.130 |
|  |  | G/T-T/T | 57 (23.6%) | 45 (33.3%) | 0.59 (0.30-1.16) |  |
|  | Recessive | G/G-G/T | 240 (99.2%) | 134 (99.3%) | 1.00 | 0.720 |
|  |  | T/T | 2 (0.8%) | 1 (0.7%) | 2.00 (0.04-90.11) |  |
|  | Over-dominant | G/G-T/T | 187 (77.3%) | 91 (67.4%) | 1.00 | 0.110 |
|  |  | G/T | 55 (22.7%) | 44 (32.6%) | 0.57 (0.29-1.13) |  |
|  | Log-additive | --- | --- | --- | 0.64 (0.34-1.20) | 0.160 |
| rs12142787 | Allele | G | 372 (77.2%) | 191 (71.8%) | 1.00 | 0.103 |
|  |  | A | 110 (22.8%) | 75 (28.2%) | 0.75 (0.54-1.06) |  |
|  | Codominant | G/G | 144 (59.8%) | 66 (49.6%) | 1.00 | 0.250 |
|  |  | A/G | 84 (34.9%) | 59 (44.4%) | 0.58 (0.30-1.11) |  |
|  |  | A/A | 13 (5.4%) | 8 (6%) | 0.75 (0.18-3.09) |  |
|  | Dominant | G/G | 144 (59.8%) | 66 (49.6%) | 1.00 | 0.110 |
|  |  | A/G-A/A | 97 (40.2%) | 67 (50.4%) | 0.59 (0.31-1.12) |  |
|  | Recessive | G/G-A/G | 228 (94.6%) | 125 (94%) | 1.00 | 0.930 |
|  |  | A/A | 13 (5.4%) | 8 (6%) | 0.94 (0.23-3.79) |  |
|  | Over-dominant | G/G-A/A | 157 (65.2%) | 74 (55.6%) | 1.00 | 0.110 |
|  |  | A/G | 84 (34.9%) | 59 (44.4%) | 0.59 (0.31-1.13) |  |
|  | Log-additive | --- | --- | --- | 0.69 (0.41-1.17) | 0.170 |
| rs3766197 | Allele | C | 407 (84.1%) | 220 (81.5%) | 1.00 | 0.359 |
|  |  | T | 77 (15.9%) | 50 (18.5%) | 0.83 (0.56-1.23) |  |
|  | Codominant | C/C | 171 (70.7%) | 91 (67.4%) | 1.00 | 0.970 |
|  |  | T/C | 65 (26.9%) | 38 (28.1%) | 0.93 (0.47-1.85) |  |
|  |  | T/T | 6 (2.5%) | 6 (4.4%) | 1.09 (0.21-5.68) |  |
|  | Dominant | C/C | 171 (70.7%) | 91 (67.4%) | 1.00 | 0.880 |
|  |  | T/C-T/T | 71 (29.3%) | 44 (32.6%) | 0.95 (0.49-1.83) |  |
|  | Recessive | C/C-T/C | 236 (97.5%) | 129 (95.6%) | 1.00 | 0.900 |
|  |  | T/T | 6 (2.5%) | 6 (4.4%) | 1.11 (0.22-5.72) |  |
|  | Over-dominant | C/C-T/T | 177 (73.1%) | 97 (71.8%) | 1.00 | 0.840 |
|  |  | T/C | 65 (26.9%) | 38 (28.1%) | 0.93 (0.47-1.83) |  |
|  | Log-additive | --- | --- | --- | 0.98 (0.56-1.71) | 0.930 |
| **ER** |  |  | **Positive** | **Negative** |  |  |
| rs2297813 | Allele | G | 626 (86.5%) | 272 (84.5%) | 1.00 | 0.394 |
|  |  | T | 98 (13.5%) | 50 (15.5%) | 0.85 (0.59-1.23) |  |
|  | Codominant | G/G | 270 (74.6%) | 113 (70.2%) | 1.00 | 0.480 |
|  |  | G/T | 86 (23.8%) | 46 (28.6%) | 0.78 (0.51-1.19) |  |
|  |  | T/T | 6 (1.7%) | 2 (1.2%) | 1.24 (0.25-6.24) |  |
|  | Dominant | G/G | 270 (74.6%) | 113 (70.2%) | 1.00 | 0.290 |
|  |  | G/T-T/T | 92 (25.4%) | 48 (29.8%) | 0.80 (0.53-1.21) |  |
|  | Recessive | G/G-G/T | 356 (98.3%) | 159 (98.8%) | 1.00 | 0.730 |
|  |  | T/T | 6 (1.7%) | 2 (1.2%) | 1.32 (0.26-6.64) |  |
|  | Over-dominant | G/G-T/T | 276 (76.2%) | 115 (71.4%) | 1.00 | 0.240 |
|  |  | G/T | 86 (23.8%) | 46 (28.6%) | 0.78 (0.51-1.18) |  |
|  | Log-additive | --- | --- | --- | 0.84 (0.58-1.23) | 0.370 |
| rs12142787 | Allele | G | 536 (74.4%) | 240 (75.0%) | 1.00 | 0.849 |
|  |  | A | 184 (25.6%) | 80 (25.0%) | 1.03 (0.76-1.40) |  |
|  | Codominant | G/G | 205 (56.9%) | 86 (53.8%) | 1.00 | 0.070 |
|  |  | A/G | 126 (35%) | 68 (42.5%) | 0.77 (0.52-1.15) |  |
|  |  | A/A | 29 (8.1%) | 6 (3.8%) | 2.04 (0.81-5.09) |  |
|  | Dominant | G/G | 205 (56.9%) | 86 (53.8%) | 1.00 | 0.500 |
|  |  | A/G-A/A | 155 (43.1%) | 74 (46.2%) | 0.88 (0.60-1.28) |  |
|  | Recessive | G/G-A/G | 331 (91.9%) | 154 (96.2%) | 1.00 | 0.055 |
|  |  | A/A | 29 (8.1%) | 6 (3.8%) | 2.26 (0.92-5.58) |  |
|  | Over-dominant | G/G-A/A | 234 (65.0%) | 92 (57.5%) | 1.00 | 0.100 |
|  |  | A/G | 126 (35%) | 68 (42.5%) | 0.72 (0.49-1.06) |  |
|  | Log-additive | --- | --- | --- | 1.03 (0.76-1.40) | 0.840 |
| rs3766197 | Allele | C | 596 (82.3%) | 273 (84.8%) | 1.00 | 0.327 |
|  |  | T | 128 (17.7%) | 49 (15.2%) | 1.20 (0.84-1.71) |  |
|  | Codominant | C/C | 248 (68.5%) | 116 (72%) | 1.00 | 0.620 |
|  |  | T/C | 100 (27.6%) | 41 (25.5%) | 1.13 (0.73-1.73) |  |
|  |  | T/T | 14 (3.9%) | 4 (2.5%) | 1.61 (0.52-5.02) |  |
|  | Dominant | C/C | 248 (68.5%) | 116 (72%) | 1.00 | 0.450 |
|  |  | T/C-T/T | 114 (31.5%) | 45 (27.9%) | 1.17 (0.77-1.76) |  |
|  | Recessive | C/C-T/C | 348 (96.1%) | 157 (97.5%) | 1.00 | 0.420 |
|  |  | T/T | 14 (3.9%) | 4 (2.5%) | 1.56 (0.50-4.83) |  |
|  | Over-dominant | C/C-T/T | 262 (72.4%) | 120 (74.5%) | 1.00 | 0.650 |
|  |  | T/C | 100 (27.6%) | 41 (25.5%) | 1.10 (0.72-1.69) |  |
|  | Log-additive | --- | --- | --- | 1.18 (0.83-1.68) | 0.370 |
| **PR** |  |  | **Positive** | **Negative** |  |  |
| rs2297813 | Allele | G | 538 (85.9%) | 359 (85.9%) | 1.00 | 0.979 |
|  |  | T | 88 (14.1%) | 59 (14.1%) | 1.00 (0.70-1.42) |  |
|  | Codominant | G/G | 231 (73.8%) | 152 (72.7%) | 1.00 | 0.610 |
|  |  | G/T | 76 (24.3%) | 55 (26.3%) | 0.91 (0.60-1.36) |  |
|  |  | T/T | 6 (1.9%) | 2 (1.0%) | 1.94 (0.38-9.78) |  |
|  | Dominant | G/G | 231 (73.8%) | 152 (72.7%) | 1.00 | 0.780 |
|  |  | G/T-T/T | 82 (26.2%) | 57 (27.3%) | 0.94 (0.63-1.41) |  |
|  | Recessive | G/G-G/T | 307 (98.1%) | 207 (99.0%) | 1.00 | 0.380 |
|  |  | T/T | 6 (1.9%) | 2 (1.0%) | 1.99 (0.39-9.99) |  |
|  | Over-dominant | G/G-T/T | 237 (75.7%) | 154 (73.7%) | 1.00 | 0.600 |
|  |  | G/T | 76 (24.3%) | 55 (26.3%) | 0.90 (0.60-1.34) |  |
|  | Log-additive | --- | --- | --- | 0.99 (0.69-1.43) | 0.970 |
| rs12142787 | Allele | G | 464 (74.6%) | 312 (75.0%) | 1.00 | 0.884 |
|  |  | A | 158 (25.4%) | 104 (25.0%) | 1.02 (0.77-1.36) |  |
|  | Codominant | G/G | 176 (56.6%) | 115 (55.3%) | 1.00 | 0.520 |
|  |  | A/G | 112 (36.0%) | 82 (39.4%) | 0.89 (0.61-1.30) |  |
|  |  | A/A | 23 (7.4%) | 11 (5.3%) | 1.38 (0.64-2.95) |  |
|  | Dominant | G/G | 176 (56.6%) | 115 (55.3%) | 1.00 | 0.780 |
|  |  | A/G-A/A | 135 (43.4%) | 93 (44.7%) | 0.95 (0.66-1.36) |  |
|  | Recessive | G/G-A/G | 288 (92.6%) | 197 (94.7%) | 1.00 | 0.330 |
|  |  | A/A | 23 (7.4%) | 11 (5.3%) | 1.44 (0.68-3.04) |  |
|  | Over-dominant | G/G-A/A | 199 (64.0%) | 126 (60.6%) | 1.00 | 0.440 |
|  |  | A/G | 112 (36.0%) | 82 (39.4%) | 0.86 (0.60-1.25) |  |
|  | Log-additive | --- | --- | --- | 1.02 (0.77-1.37) | 0.870 |
| rs3766197 | Allele | C | 512 (81.8%) | 355 (84.9%) | 1.00 | 0.185 |
|  |  | T | 144 (18.2%) | 63 (15.1%) | 1.26 (0.90-1.76) |  |
|  | Codominant | C/C | 213 (68.0%) | 150 (71.8%) | 1.00 | 0.260 |
|  |  | T/C | 86 (27.5%) | 55 (26.3%) | 1.07 (0.72-1.60) |  |
|  |  | T/T | 14 (4.5%) | 4 (1.9%) | 2.43 (0.78-7.56) |  |
|  | Dominant | C/C | 213 (68.0%) | 150 (71.8%) | 1.00 | 0.440 |
|  |  | T/C-T/T | 100 (31.9%) | 59 (28.2%) | 1.16 (0.79-1.71) |  |
|  | Recessive | C/C-T/C | 299 (95.5%) | 205 (98.1%) | 1.00 | 0.110 |
|  |  | T/T | 14 (4.5%) | 4 (1.9%) | 2.38 (0.77-7.38) |  |
|  | Over-dominant | C/C-T/T | 227 (72.5%) | 154 (73.7%) | 1.00 | 0.870 |
|  |  | T/C | 86 (27.5%) | 55 (26.3%) | 1.03 (0.69-1.54) |  |
|  | Log-additive | --- | --- | --- | 1.22 (0.87-1.70) | 0.240 |
| **Her-2** |  |  | **Positive** | **Negative** |  |  |
| rs2297813 | Allele | G | 212 (84.1%) | 334 (86.1%) | 1.00 | 0.495 |
|  |  | T | 40 (15.9%) | 54 (13.9%) | 1.17 (0.75-1.82) |  |
|  | Codominant | G/G | 89 (70.6%) | 142 (73.2%) | 1.00 | 0.630 |
|  |  | G/T | 34 (27.0%) | 50 (25.8%) | 1.11 (0.66-1.85) |  |
|  |  | T/T | 3 (2.4%) | 2 (1.0%) | 2.31 (0.37-14.25) |  |
|  | Dominant | G/G | 89 (70.6%) | 142 (73.2%) | 1.00 | 0.570 |
|  |  | G/T-T/T | 37 (29.4%) | 52 (26.8%) | 1.15 (0.70-1.91) |  |
|  | Recessive | G/G-G/T | 123 (97.6%) | 192 (99.0%) | 1.00 | 0.380 |
|  |  | T/T | 3 (2.4%) | 2 (1%) | 2.24 (0.37-13.76) |  |
|  | Over-dominant | G/G-T/T | 92 (73.0%) | 144 (74.2%) | 1.00 | 0.750 |
|  |  | G/T | 34 (27.0%) | 50 (25.8%) | 1.09 (0.65-1.81) |  |
|  | Log-additive | --- | --- | --- | 1.19 (0.75-1.88) | 0.460 |
| rs12142787 | Allele | G | 182 (72.8%) | 296 (76.7%) | 1.00 | 0.268 |
|  |  | A | 68 (27.2%) | 90 (23.3%) | 1.23 (0.85-1.77) |  |
|  | Codominant | G/G | 66 (52.8%) | 114 (59.1%) | 1.00 | 0.420 |
|  |  | A/G | 50 (40.0%) | 68 (35.2%) | 1.34 (0.83-2.18) |  |
|  |  | A/A | 9 (7.2%) | 11 (5.7%) | 1.48 (0.58-3.77) |  |
|  | Dominant | G/G | 66 (52.8%) | 114 (59.1%) | 1.00 | 0.190 |
|  |  | A/G-A/A | 59 (47.2%) | 79 (40.9%) | 1.36 (0.86-2.17) |  |
|  | Recessive | G/G-A/G | 116 (92.8%) | 182 (94.3%) | 1.00 | 0.570 |
|  |  | A/A | 9 (7.2%) | 11 (5.7%) | 1.31 (0.52-3.26) |  |
|  | Over-dominant | G/G-A/A | 75 (60.0%) | 125 (64.8%) | 1.00 | 0.300 |
|  |  | A/G | 50 (40.0%) | 68 (35.2%) | 1.29 (0.80-2.07) |  |
|  | Log-additive | --- | --- | --- | 1.27 (0.88-1.85) | 0.200 |
| rs3766197 | Allele | C | 216 (85.7%) | 323 (83.2%) | 1.00 | 0.403 |
|  |  | T | 36 (14.3%) | 65 (16.8%) | 0.83 (0.53-1.29) |  |
|  | Codominant | C/C | 93 (73.8%) | 135 (69.6%) | 1.00 | 0.660 |
|  |  | T/C | 30 (23.8%) | 53 (27.3%) | 0.80 (0.48-1.36) |  |
|  |  | T/T | 3 (2.4%) | 6 (3.1%) | 0.72 (0.17-2.95) |  |
|  | Dominant | C/C | 93 (73.8%) | 135 (69.6%) | 1.00 | 0.370 |
|  |  | T/C-T/T | 33 (26.2%) | 59 (30.4%) | 0.80 (0.48-1.32) |  |
|  | Recessive | C/C-T/C | 123 (97.6%) | 188 (96.9%) | 1.00 | 0.700 |
|  |  | T/T | 3 (2.4%) | 6 (3.1%) | 0.76 (0.19-3.11) |  |
|  | Over-dominant | C/C-T/T | 96 (76.2%) | 141 (72.7%) | 1.00 | 0.440 |
|  |  | T/C | 30 (23.8%) | 53 (27.3%) | 0.81 (0.48-1.37) |  |
|  | Log-additive | --- | --- | --- | 0.82 (0.53-1.27) | 0.370 |
| **Ki67** |  |  | **> 25%** | **≤ 25%** |  |  |
| rs2297813 | Allele | G | 615 (85.7%) | 260 (86.7%) | 1.00 | 0.672 |
|  |  | T | 103 (14.3%) | 40 (13.3%) | 1.09 (0.73-1.61) |  |
|  | Codominant | G/G | 259 (72.1%) | 114 (76.0%) | 1.00 | 0.140 |
|  |  | G/T | 97 (27.0%) | 32 (21.3%) | 1.31 (0.83-2.09) |  |
|  |  | T/T | 3 (0.8%) | 4 (2.7%) | 0.31 (0.07-1.42) |  |
|  | Dominant | G/G | 259 (72.1%) | 114 (76.0%) | 1.00 | 0.420 |
|  |  | G/T-T/T | 100 (27.9%) | 36 (24.0%) | 1.20 (0.77-1.87) |  |
|  | Recessive | G/G-G/T | 356 (99.2%) | 146 (97.3%) | 1.00 | 0.110 |
|  |  | T/T | 3 (0.8%) | 4 (2.7%) | 0.29 (0.06-1.32) |  |
|  | Over-dominant | G/G-T/T | 262 (73.0%) | 118 (78.7%) | 1.00 | 0.200 |
|  |  | G/T | 97 (27.0%) | 32 (21.3%) | 1.35 (0.85-2.13) |  |
|  | Log-additive | --- | --- | --- | 1.07 (0.71-1.61) | 0.740 |
| rs12142787 | Allele | G | 535 (75.1%) | 223 (74.3%) | 1.00 | 0.787 |
|  |  | A | 177 (24.9%) | 77 (25.7%) | 0.96 (0.70-1.31) |  |
|  | Codominant | G/G | 198 (55.6%) | 87 (58.0%) | 1.00 | 0.160 |
|  |  | A/G | 139 (39.0%) | 49 (32.7%) | 1.26 (0.83-1.92) |  |
|  |  | A/A | 19 (5.3%) | 14 (9.3%) | 0.61 (0.29-1.28) |  |
|  | Dominant | G/G | 198 (55.6%) | 87 (58.0%) | 1.00 | 0.590 |
|  |  | A/G-A/A | 158 (44.4%) | 63 (42.0%) | 1.12 (0.75-1.65) |  |
|  | Recessive | G/G-A/G | 337 (94.7%) | 136 (90.7%) | 1.00 | 0.120 |
|  |  | A/A | 19 (5.3%) | 14 (9.3%) | 0.55 (0.27-1.15) |  |
|  | Over-dominant | G/G-A/A | 217 (61.0%) | 101 (67.3%) | 1.00 | 0.160 |
|  |  | A/G | 139 (39.0%) | 49 (32.7%) | 1.34 (0.89-2.01) |  |
|  | Log-additive | --- | --- | --- | 0.97 (0.71-1.32) | 0.830 |
| rs3766197 | Allele | C | 595 (82.9%) | 252 (84.0%) | 1.00 | 0.60 |
|  |  | T | 123 (17.1%) | 48 (16.0%) | 1.09 (0.75-1.56) |  |
|  | Codominant | C/C | 249 (69.4%) | 106 (70.7%) | 1.00 | 0.940 |
|  |  | T/C | 97 (27.0%) | 40 (26.7%) | 0.98 (0.63-1.52) |  |
|  |  | T/T | 13 (3.6%) | 4 (2.7%) | 1.22 (0.38-3.89) |  |
|  | Dominant | C/C | 249 (69.4%) | 106 (70.7%) | 1.00 | 0.990 |
|  |  | T/C-T/T | 110 (30.6%) | 44 (29.3%) | 1.00 (0.66-1.53) |  |
|  | Recessive | C/C-T/C | 346 (96.4%) | 146 (97.3%) | 1.00 | 0.720 |
|  |  | T/T | 13 (3.6%) | 4 (2.7%) | 1.23 (0.39-3.88) |  |
|  | Over-dominant | C/C-T/T | 262 (73.0%) | 110 (73.3%) | 1.00 | 0.900 |
|  |  | T/C | 97 (27.0%) | 40 (26.7%) | 0.97 (0.63-1.50) |  |
|  | Log-additive | --- | --- | --- | 1.02 (0.71-1.47) | 0.900 |
| **LNM** |  |  | **Yes** | **No** |  |  |
| rs2297813 | Allele | G | 334 (86.1%) | 415 (86.1%) | 1.00 | 0.990 |
|  |  | T | 54 (13.9%) | 67 (13.9%) | 1.00 (0.68-1.47) |  |
|  | Codominant | G/G | 143 (73.7%) | 177 (73.4%) | 1.00 | 0.950 |
|  |  | G/T | 48 (24.7%) | 61 (25.3%) | 0.98 (0.63-1.53) |  |
|  |  | T/T | 3 (1.6%) | 3 (1.2%) | 1.29 (0.25-6.55) |  |
|  | Dominant | G/G | 143 (73.7%) | 177 (73.4%) | 1.00 | 0.990 |
|  |  | G/T-T/T | 51 (26.3%) | 64 (26.6%) | 1.00 (0.65-1.53) |  |
|  | Recessive | G/G-G/T | 191 (98.5%) | 238 (98.8%) | 1.00 | 0.760 |
|  |  | T/T | 3 (1.6%) | 3 (1.2%) | 1.29 (0.26-6.55) |  |
|  | Over-dominant | G/G-T/T | 146 (75.3%) | 180 (74.7%) | 1.00 | 0.920 |
|  |  | G/T | 48 (24.7%) | 61 (25.3%) | 0.98 (0.63-1.52) |  |
|  | Log-additive | --- | --- | --- | 1.01 (0.68-1.51) | 0.950 |
| rs12142787 | Allele | G | 296 (76.3%) | 350 (73.5%) | 1.00 | 0.350 |
|  |  | A | 92 (23.7%) | 126 (26.5%) | 0.86 (0.63-1.18) |  |
|  | Codominant | G/G | 112 (57.7%) | 133 (55.9%) | 1.00 | 0.330 |
|  |  | A/G | 72 (37.1%) | 84 (35.3%) | 1.01 (0.67-1.51) |  |
|  |  | A/A | 10 (5.2%) | 21 (8.8%) | 0.56 (0.25-1.24) |  |
|  | Dominant | G/G | 112 (57.7%) | 133 (55.9%) | 1.00 | 0.670 |
|  |  | A/G-A/A | 82 (42.3%) | 105 (44.1%) | 0.92 (0.62-1.35) |  |
|  | Recessive | G/G-A/G | 184 (94.8%) | 217 (91.2%) | 1.00 | 0.130 |
|  |  | A/A | 10 (5.2%) | 21 (8.8%) | 0.56 (0.26-1.22) |  |
|  | Over-dominant | G/G-A/A | 122 (62.9%) | 154 (64.7%) | 1.00 | 0.730 |
|  |  | A/G | 72 (37.1%) | 84 (35.3%) | 1.07 (0.72-1.60) |  |
|  | Log-additive | --- | --- | --- | 0.86 (0.63-1.17) | 0.340 |
| rs3766197 | Allele | C | 325 (83.8%) | 406 (84.2%) | 1.00 | 0.850 |
|  |  | T | 63 (16.2%) | 76 (15.8%) | 1.04 (0.72-1.49) |  |
|  | Codominant | C/C | 138 (71.1%) | 173 (71.8%) | 1.00 | 0.980 |
|  |  | T/C | 49 (25.3%) | 60 (24.9%) | 1.01 (0.65-1.57) |  |
|  |  | T/T | 7 (3.6%) | 8 (3.3%) | 1.10 (0.39-3.12) |  |
|  | Dominant | C/C | 138 (71.1%) | 173 (71.8%) | 1.00 | 0.930 |
|  |  | T/C-T/T | 56 (28.9%) | 68 (28.2%) | 1.02 (0.67-1.55) |  |
|  | Recessive | C/C-T/C | 187 (96.4%) | 233 (96.7%) | 1.00 | 0.860 |
|  |  | T/T | 7 (3.6%) | 8 (3.3%) | 1.10 (0.39-3.10) |  |
|  | Over-dominant | C/C-T/T | 145 (74.7%) | 181 (75.1%) | 1.00 | 0.980 |
|  |  | T/C | 49 (25.3%) | 60 (24.9%) | 1.00 (0.65-1.56) |  |
|  | Log-additive | --- | --- | --- | 1.03 (0.72-1.46) | 0.890 |

OR: Odds ratio; 95% CI: 95% confidence interval; ER: Estrogen receptor; PR: Progesterone receptor; Her-2: Human epidermal growth factor receptor 2; LNM: Lymph node metastasis
